# A new laboratory evolution approach to select for constitutive acetic acid tolerance in *Saccharomyces cerevisiae* and identification of causal mutations

**DOI:** 10.1186/s13068-016-0583-1

**Published:** 2016-08-12

**Authors:** Daniel González-Ramos, Arthur R. Gorter de Vries, Sietske S. Grijseels, Margo C. van Berkum, Steve Swinnen, Marcel van den Broek, Elke Nevoigt, Jean-Marc G. Daran, Jack T. Pronk, Antonius J. A. van Maris

**Affiliations:** 1 Department of Biotechnology, Delft University of Technology, Julianalaan 67, 2628 BC Delft, The Netherlands; 2 Department of Life Sciences and Chemistry, Jacobs University Bremen gGmbH, Campus Ring 1, 28759 Bremen, Germany

**Keywords:** Yeast, Acetate, Evolutionary engineering, Bioethanol, Inhibitors

## Abstract

**Background:**

Acetic acid, released during hydrolysis of lignocellulosic feedstocks for second generation bioethanol production, inhibits yeast growth and alcoholic fermentation. Yeast biomass generated in a propagation step that precedes ethanol production should therefore express a high and constitutive level of acetic acid tolerance before introduction into lignocellulosic hydrolysates. However, earlier laboratory evolution strategies for increasing acetic acid tolerance of *Saccharomyces cerevisiae*, based on prolonged cultivation in the presence of acetic acid, selected for inducible rather than constitutive tolerance to this inhibitor.

**Results:**

Preadaptation in the presence of acetic acid was shown to strongly increase the fraction of yeast cells that could initiate growth in the presence of this inhibitor. Serial microaerobic batch cultivation, with alternating transfers to fresh medium with and without acetic acid, yielded evolved *S. cerevisiae* cultures with constitutive acetic acid tolerance. Single-cell lines isolated from five such evolution experiments after 50–55 transfers were selected for further study. An additional constitutively acetic acid tolerant mutant was selected after UV-mutagenesis. All six mutants showed an increased fraction of growing cells upon a transfer from a non-stressed condition to a medium containing acetic acid. Whole-genome sequencing identified six genes that contained (different) mutations in multiple acetic acid-tolerant mutants. Haploid segregation studies and expression of the mutant alleles in the unevolved ancestor strain identified causal mutations for the acquired acetic acid tolerance in four genes (*ASG1*, *ADH3*, *SKS1* and *GIS4*). Effects of the mutations in *ASG1*, *ADH3* and *SKS1* on acetic acid tolerance were additive.

**Conclusions:**

A novel laboratory evolution strategy based on alternating cultivation cycles in the presence and absence of acetic acid conferred a selective advantage to constitutively acetic acid-tolerant mutants and may be applicable for selection of constitutive tolerance to other stressors. Mutations in four genes (*ASG1*, *ADH3*, *SKS1* and *GIS4*) were identified as causative for acetic acid tolerance. The laboratory evolution strategy as well as the identified mutations can contribute to improving acetic acid tolerance in industrial yeast strains.

**Electronic supplementary material:**

The online version of this article (doi:10.1186/s13068-016-0583-1) contains supplementary material, which is available to authorized users.

## Background

Second generation bioethanol production uses lignocellulosic material from forestry residues, agricultural residues or energy crops as feedstocks. Use of these substrates is considered advantageous because of their abundance, availability, low cost and complementarity with food production [1–3]. Current processes for conversion of lignocellulosic biomass involve pretreatment of the substrate to disrupt its structure, followed by enzymatic hydrolysis to release monomeric sugars. The subsequent fermentation of these sugars to ethanol almost exclusively relies on the yeast *Saccharomyces cerevisiae* [4–7]. Hydrolysis not only releases fermentable sugars, but also furans, phenols and weak acids, whose presence can negatively affect yeast growth and ethanol production [8, 9].

Acetic acid, released during de-acetylation of hemicellulose, is the most abundant weak acid in lignocellulosic hydrolysates, at concentrations that can exceed 10 g/L [10]. Inoculation into acetic acid containing media can cause a reduction in the specific growth rate and biomass yield of *S. cerevisiae*, as well as a substantially increased lag phase [11–13]. To minimize the risk of bacterial contamination and the requirement for base addition, alcoholic fermentation is preferably carried out at low pH, which enhances the toxicity of acetic acid [14–16]. At pH values below its dissociation constant (p*K*a = 4.76), acetic acid predominantly occurs in its protonated form, which can enter cells via passive diffusion [12, 16]. In the near-neutral cytosol, the acid dissociates into the acetate anion and a proton [17]. To prevent intracellular acidification, yeast cells expel protons via the plasma membrane H^+^-ATPase Pma1 [11, 18–20]. The resulting diversion of ATP from cellular growth and maintenance is an important contributor to the inhibitory effects of acetic acid on *S. cerevisiae* [11]. Additionally, intracellular accumulation of acetate anions can contribute to inhibition of specific cellular processes [21], osmotic stress, and in aerobic cultures, oxidative stress [22–24].

Upon transfer of *S. cerevisiae* cultures from a medium without acetic acid to a medium with an inhibitory concentration of acetic acid, only a small fraction of the population is able to resume growth [25]. The fraction of cells able to grow decreases with increasing acetic acid concentration and is strain dependent [25, 26]. This culture heterogeneity contributes to a latency phase, defined as the time that elapses before growth is observed spectrophotometrically in an acetic acid containing medium. The fraction of a non-stressed population that is able to grow upon transfer to a medium with acetic acid is therefore a relevant measure for acetic acid tolerance of different strains [25]. The actual length of the latency phase is further influenced by the time that this fraction of cells needs to start growing, and by their specific growth rate [20, 25].

To mitigate inhibitory effects of acetic acid in lignocellulosic hydrolysates, development of yeast strains with increased tolerance would be highly beneficial. Several previous studies aimed to understand and improve the response of *S. cerevisiae* to acetic acid. Transcriptome analysis identified a large number of genes that are differentially expressed upon exposure to acetic acid stress. These genes encode proteins involved in functions, such as transcription control, internal pH homeostasis, carbohydrate metabolism, cell-wall assembly, and biogenesis of mitochondria, ribosomes and the vacuole [27, 28]. However, despite clear progress, the underlying mechanisms of acetic acid tolerance remain incompletely understood [29], which hinders knowledge-based engineering strategies.

Natural and induced diversity of acetic acid tolerance among *S. cerevisiae* strains has also been explored to investigate its genetic basis. For example, generation of haploid segregants from crosses of highly tolerant and less tolerant strains, followed by quantitative-trait-locus (QTL) analysis, enabled the identification of multiple alleles that affect acetic acid tolerance in this yeast [30]. Increased acetic acid tolerance has also been reported for strains carrying targeted genetic modifications, such as overexpression of *TAL1,* encoding a transaldolase [31], overexpression of *PEP3*, encoding a protein subunit involved in vacuolar biogenesis [32], overexpression of the transcription factor *HAA1* [33], introduction of an artificial zinc-finger based transcription factor [34] and introduction of an ascorbic-acid production pathway [35]. However, the levels of tolerance reached by these approaches are not sufficient for the high concentrations of acetic acid present in lignocellulosic hydrolysates at industrially relevant pH values.

Laboratory evolution allows the selection of specific phenotypes without the requirement for in-depth understanding of the underlying mechanisms [9, 36, 37] and has been successfully used to improve the tolerance of yeast to butanol [38] and other stresses, such as freezing–thawing cycles, increased temperature, ethanol, and oxidative stress [39]. This approach has also been used to improve acetic acid tolerance in bacteria [40] and yeast. Wright et al. [41] used repeated batch cultivation at progressively increasing concentrations of acetic acid to select a *S. cerevisiae* strain with a higher acid–acid tolerance. However, after growing the evolved strain in the absence of acetic acid, the strain was no longer able to grow at high acetic acid concentrations. Although the strain was genetically stable, increased tolerance to acetic acid could only be shown after pre-cultivation in the presence of acetic acid. These observations indicated that the increased tolerance of the evolved strain was not constitutive but required induction by acetic acid [41]. In another study, induction of acetic acid tolerance was shown to occur upon exposure of cells to low levels of acetic acid, resulting in a reversible acetic acid tolerant phenotype [42]. In accordance with the proposed role of cytosolic pH in acetic acid tolerance, these adapted cells showed a less dramatic decrease in intracellular pH upon exposure to acetic acid compared to non-adapted cells upon exposure to acetic acid [11, 26]. Preadaptation to acetic acid was also shown to protect yeast cells from acetic acid-mediated programmed cell death [43]. To benefit from such an inducible acetic acid tolerance in industrial processes, an adaptation step would have to be implemented prior to the fermentation, making the whole process more complex. Yeast strains with constitutively high-level tolerances, which do not require adaptation, would therefore clearly be preferable for industrial applications.

The aim of this study is to investigate how laboratory evolution and mutagenesis can be used to obtain *S. cerevisiae* strains with constitutive acetic acid tolerance. To maximize the selective pressure on constitutively tolerant strains and simultaneously minimize the competitiveness of cells with inducible acetic acid tolerance, laboratory evolution was performed by alternatingly growing *S. cerevisiae* cells in medium with and without acetic acid. In parallel, mutagenesis and screening was applied to obtain constitutively tolerant mutants without laboratory evolution. Subsequently, a combination of whole-genome sequencing and crossing and segregation studies was used to investigate the mutations underlying the constitutively acetic acid tolerant phenotype. The impact of these mutations on acetic acid tolerance was confirmed by reverse engineering the evolved phenotype into the unevolved reference strain.

## Methods

### Strains, media and growth conditions

The haploid laboratory strain *S. cerevisiae* CEN.PK113-7D was used as the reference strain in all experiments [44, 45]. Related yeast strains, generated and used in this study, are listed in Tables 1 and 2. Unless stated otherwise, yeast cultures were grown in 500-mL shake-flasks containing 100 mL synthetic medium with 20 g/L glucose as the sole carbon and energy source (SMG), at 30 °C and 200 rpm. Synthetic medium was prepared according to Verduyn et al. [46], with the following modifications: ammonium sulphate (5 g/L) was replaced by 2.3 g/L urea to prevent acidification due to nitrogen source consumption and 6.6 g/L K_2_SO_4_ was added [47]. The anaerobic growth factors ergosterol and Tween-80 were added to all cultures in this study as previously described [48]. Unless stated, otherwise, for media without acetic acid, the pH was set to 6 with KOH and media were filter sterilized. Media with acetic acid concentrations ranging from 0 to 15 g/L were prepared by mixing SMG and SMG containing 15 g/L acetic acid (both set at pH 4.5) in different proportions. SMG agar plates containing 9 g/L acetic acid were prepared by mixing equal volumes of two solutions: (1) distilled water containing 40 g/L of agar at pH 4.5, autoclaved at 121 °C for 20 min, and (2) filter sterilized double-strength SMG, containing 18 g/L acetic acid and set at pH 4.5. Selection of strains transformed with kanMX, natMX or hphNT1 marker genes was performed on YPD agar plates (containing 10 g/L yeast extract, 20 g/L peptone, 20 g/L glucose and 20 g/L agar) supplemented with 200 μg/mL G418 (InvivoGen, San Diego, CA), 100 µg/mL nourseothricin (Hans-Knoll Institute für Naturstoff-Forschung, Jena, Germany), or 200 μg/mL hygromycin (Life technologies, Carlsbad, CA), respectively. Counter selection of *GIN11M86* [49] was performed on YPGal agar plates containing 10 g/L yeast extract, 20 g/L peptone, 20 g/L galactose and 20 g/L agar. Selection of diploid strains was performed on SMG agar plates without uracil [46] supplemented with G418. Sporulation was performed according to Bahalul et al. [50]; after growth of diploid strains in YPA (yeast extract 10 g/L, peptone 20 g/L, potassium acetate 10 g/L) for 2 days, cells were washed with water and transferred to a solution of 20 g/L potassium acetate at pH 7.0. After 2–3 days of incubation, spores where plated on SMG and single colonies were isolated.

**Table 1:** Acetic acid tolerant mutants derived from CEN.PK113-7D through evolution/mutagenesis and crossing

Strain	Collection name	Relevant description	Source
CEN.PK113-7D		MATa	[45]
IMK439		MATα ura3∆::kanMX	[38]
MUT1A	IMS0379	Acetic acid tolerant mutant evolved from CEN.PK113-7D	This study
MUT2B	IMS0527	Acetic acid tolerant mutant evolved from CEN.PK113-7D	This study
MUT3E	IMS0528	Acetic acid tolerant mutant evolved from CEN.PK113-7D	This study
HAT1E	IMS0529	Acetic acid tolerant mutant evolved from CEN.PK113-7D	This study
HAT2A	IMS0530	Acetic acid tolerant mutant evolved from CEN.PK113-7D	This study
UV-E3	IMS0378	Acetic acid tolerant UV-radiated mutant from CEN.PK113-7D	This study
MUT1A-D	IMS0531	Diploid obtained from crossing MUT1A with IMK439	This study
MUT2B-D	IMS0532	Diploid obtained from crossing MUT2B with IMK439	This study
MUT3E-D	IMS0391	Diploid obtained from crossing MUT3E with IMK439	This study
HAT1E-D	IMS0392	Diploid obtained from crossing HAT1E with IMK439	This study
HAT2A-D	IMS0402	Diploid obtained from crossing HAT2A with IMK439	This study

IMK439 was used for mating experiments, which is congenic with CEN.PK113-7D, but has an opposite mating type and contains a URA3 deletion as well as the kanMX marker. The haploid segregants obtained by sporulation of the diploid strains are shown in Additional file 9

**Table 2:** Engineered *S. cerevisiae* strains constructed in this study by replacement, in the reference strain CEN.PK113-7D, of the native alleles of ASG1, ADH3, GIS4 and/or SKS1 by mutated alleles identified in mutagenized and/or evolved acetic acid tolerant strains

Strain name	Collection name	Origin of mutation	Genotype
{ASG1}^MUT1A^	IMI317	MUT1A	MATa, asg1^G1248A^
{ASG1,ADH3}^MUT1A^	IMI318	MUT1A	MATa, asg1^G1248A^, adh3^G416T^
{ASG1}^MUT2B^	IMI320	MUT2B	MATa, asg1^G1248T^
{ASG1,ADH3}^MUT2B^	IMI319	MUT2B	MATa, asg1^G1248T^, adh3^T966G^
{ASG1,ADH3,SKS1}^MUT2B^	IMI327	MUT2B	MATa, asg1^G1248T^, adh3^T966G^, sks1^G821T^
{GIS4}^MUT3E^	IMI307	MUT3E	MATa, gis4^G1322C^
{ASG1}^HAT1E^	IMI316	HAT1E	MATa, asg1^A1979G^
{ASG1}^HAT2A^	IMI321	HAT2A	MATa, asg1^G2881C^
{ASG1,ADH3}^HAT2A^	IMI326	HAT2A	MATa, asg1^G2881C^, adh3^T201A^
{ASG1,ADH3,SKS1}^HAT2A^	IMI328	HAT2A	MATa, asg1^G2881C^, adh3^T201A^, sks1^C617A^
{GIS4}^UV-E3^	IMI308	UV-E3	MATa, gis4^G295A^

### Laboratory evolution

Repeated batch cultivation of *S. cerevisiae* CEN.PK113-7D was performed in a simple microaerobic cultivation system. Cultures were grown in 30 mL serum bottles, containing 25 mL medium and closed with a butyl-rubber seal. The seal was punctured with a needle connected to a syringe to enable release of overpressure generated by CO_2_ production. These cultures were incubated at 30 °C and shaken at 200 rpm. Serial transfer was performed by alternating cultivation in SMG without acetic acid with cultivation in SMG containing increasing concentrations of acetic acid, ranging from 10 to 18 g/L. Cultures with acetic acid were inoculated by direct transfer of 1 mL from the prior bottle without acetic acid. Cultures without acetic acid were inoculated by spinning down 0.1 mL of broth from the prior culture with acetic acid, which was washed with water and resuspended in SMG prior to inoculation. Five independent evolution lines were performed (MUT1, MUT2, MUT3, HAT1 and HAT2). The HAT1 and HAT2 evolution lines were started from CEN.PK113-7D stock cultures while the MUT1, MUT2 and MUT3 evolution lines were started with UV-mutagenized CEN.PK113-7D. Before UV mutagenesis, exponentially growing shake-flask cultures on YPD were washed with water and resuspended in SMG to an OD_660_ of 1.0. Subsequently, 25 mL of the resulting cell suspension was poured in a (9 cm diameter) petri dish, shaken at 100 rpm inside a laminar-flow cabinet and irradiated with a UV lamp (TUV 30 W T8, Philips, Eindhoven, The Netherlands) at a radiation peak of 253.7 nm. Suspensions used to initiate evolution lines MUT1, MUT2 and MUT3 were irradiated with UV doses yielding survival rates of 18, 22 and 16 %, respectively. Evolution experiments MUT1 and MUT2 involved 50 transfers, while the MUT3, HAT1 and HAT2 experiments involved 55, 54 and 51 transfers, respectively. At the end of the laboratory evolution experiments, single cell lines were isolated from each evolution line by streaking on SMG agar plates.

### Mutagenesis and screening for acetic acid tolerant mutants

*Saccharomyces cerevisiae* CEN.PK113-7D was irradiated with UV light as described above, but at a dose resulting in a survival rate of 2 %. After irradiation, the cell suspension was transferred to a serum flask and grown in SMG in the dark for 2 days. 40 colonies were isolated on SMG agar plates with 9 g/L acetic acid and each transferred into one well of a 96-well microtiter plate containing 50 μL of SMG using a sterile pipet tip. Additionally, 8 wells were inoculated with the reference strain CEN.PK113-7D. The microtiter plate was then sealed with a gas impermeable seal (NUNC, Roskilde, Denmark) and incubated for 2 days in an orbital shaker (PHMP-4 Thermoshaker for microplates, Grant Instruments, Shepreth, UK) at 30 °C and 700 rpm. This initial plate was used to inoculate four 96-well plates containing 100 µL/well of SMG, two with 9 g/L acetic acid and two with 10 g/L acetic acid. These 96-well plates were sealed and incubated (30 °C, 700 rpm). After four days, the OD_660_ of all wells was measured and the five mutants with the highest OD_660_ values at each acetic acid concentrations were selected. Single-cell lines of mutants were isolated by picking a colony from YPD agar plates and growing them overnight in microaerobic shake-flasks containing 20 mL SMG, along with a similar culture of the reference strain CEN.PK113-7D. All cultures were then diluted with sterile water to an OD_660_ of 5.0 and eight 50 µL aliquots of each strain were distributed into a 96-well plate. This microtiter plate was used to inoculate a microtiter plate containing 100 µL/well of SMG with 10 g/L acetic acid using a sterile pin replicator. The plate was sealed, incubated for 4 days and the final OD_660_ was measured. The mutant with the highest average OD_660_ was named UV-E3 and further characterized for its acetic acid tolerance.

### Analysis of acetic acid tolerance: OD_660_ in stationary phase

Shake-flask cultures were grown overnight until stationary phase in SMG and diluted with sterile water to an OD_660_ of 5. 96-well plates were filled with 100 μL/well of SMG containing acetic acid concentrations ranging from 0 to 15 g/L (12 different concentrations with 8 replicas each). The plates were inoculated with a sterile pin replicator, sealed with a gas impermeable seal (NUNC, Roskilde, Denmark) and incubated at 30 °C for 5 days without shaking. After incubation, cells were resuspended in a MS2 minishaker (IKA, Staufen, Germany), seals were removed and OD_660_ of each well was measured in a GENIos Pro micro plate spectrophotometer (Tecan, Männedorf, Switzerland). Average OD_660_ and standard deviation of replicate wells were then calculated. Where indicated in the results section, glucose concentrations in culture supernatants were obtained after centrifugation and analysed via HPLC using an Aminex HPX-87H ion exchange column operated at 60 °C with 5 mM H_2_SO_4_ as mobile phase at a flow rate of 0.6 mL/min.

### Analysis of acetic acid tolerance: latency phase and growth rates

Overnight shake-flask cultures grown until stationary phase in SMG were used to inoculate 1.5-mL Eppendorf tubes containing 1 mL of SMG with acetic acid concentrations ranging from 0 to 15 g/L, at an initial OD_660_ of 0.2 for growth rate determination or of 0.1 for latency phase determination. 100 μL aliquots from each tube were distributed into different wells of a 96-well plate. The plates were sealed with a gas impermeable seal and incubated in a GENIos Pro micro plate spectrophotometer at 400 rpm for 5 days. OD_660_ was recorded at 15 min intervals. The duration of the latency phase was determined as the time after inoculation at which the OD_660_ increased above 0.12. Specific growth rates were estimated from exponential fits of the OD_660_ versus time.

### Analysis of acetic acid tolerance: fraction of cells growing in the presence of acetic acid

Overnight shake-flask cultures grown until stationary phase in SMG were diluted with sterile water to an OD_660_ of 1. 100 μL of serial tenfold dilution of these cell suspensions were plated on SMG agar plates (pH 4.5) with and without 9 g/L acetic acid. The number of colony-forming units (CFU) per mL for each dilution was calculated after 5 days incubation in a Bactron X-2E anaerobic chamber (Sheldon Manufacturing Inc., Cornelius, OR) at 30 °C. For each strain, the fraction of cells able to grow on agar plates containing acetic acid was calculated by dividing the CFU/mL counts on agar plates with acetic acid by the CFU/mL count without acetic acid.

### Whole-genome sequencing of the acetic acid tolerant mutants

Genomic DNA of the strains CEN.PK113-7D, HAT1E, HAT2A, MUT1A, MUT2B, MUT3E and UV-E3 was prepared as described previously [51]. Libraries of 300-bp inserts were constructed and paired-end sequenced (101 base pair reads) using an Illumina HISeq 2500 sequencer (Baseclear BV, Leiden, The Netherlands). A minimum data quantity of 1 GB was generated for each strain, representing a minimum 83-fold average coverage. Sequence reads of each strain were mapped onto the CEN.PK113-7D genome [44] using the Burrows–Wheeler Alignment tool (BWA) and further processed using SAMtools [52–54]. Single-nucleotide variations were determined using SAMtools’ varFilter. Default settings were used, except that the maximum read depth was set to 400X (-D400). To minimize false positive mutation calls, custom Perl scripts were used for further mutation filtering: (i) mutation calls containing ambiguous bases in mapping consensus were filtered out, (ii) only the single-nucleotide variations with a variant quality, defined as the Phred-scaled probability that the mutation call is incorrect [55, 56], of at least 20 were kept and (iii) mutations with a depth of coverage <10X were discarded. Finally, the single-nucleotide variations were positioned by their genomic locations; coding effects were predicted and functionally annotated according to the CEN.PK113-7D sequence annotation [57]. Raw sequencing data have been deposited as short-read archives (Bioproject: PRJNA313456/SRP070976, individual accession numbers in Additional file 1). The Magnolya algorithm was used to analyse copy number variation using Newbler (454 Life Sciences, Branford, CT) for co-assembly of sequence reads of CEN.PK113-7D and the sample of interest [58]. Integer copy numbers of the assembled contigs were estimated using the Poisson mixture model (PMM) algorithm in Magnolya.

### Crossing, sporulation and screening for acetic acid-tolerant haploid segregants

Mutants evolved for increased acetic acid tolerance were crossed with IMK439, a strain derived from CEN.PK113-1A (*MATα*, otherwise isogenic to CEN.PK113-7D) by replacing its *URA3* gene with the kanMX marker [38]. To this end, each mutant was grown together with IMK439 on SMG agar plates without uracil and with 200 µg/mL G418, on which only diploidized strains could grow. After sporulation, random spore isolation was performed as described previously [50] and spores were plated on SMG without uracil. Each segregant was then transferred into one well of a 96-well plate containing 50 µL of SMG using a sterile pipet tip. On every plate, the reference strain CEN.PK113-7D, the evolved acetic acid tolerant parent and the diploid strain from which the segregants were derived, were each inoculated in 8 wells as internal controls. After sealing the plate and overnight incubation at 30 °C without shaking, these plates were used to inoculate 96-well plates filled with 100 μL/well of SMG with acetic acid concentrations ranging from 9 to 15 g/L using a sterile pin replicator. After sealing with a gas impermeable seal, plates were incubated at 30 °C for 5 days without shaking, followed by measurement of OD_660_. For each mutant, ten segregants with the same tolerance as the evolved tolerant mutant and ten segregants with the same sensitivity as CEN.PK113-7D were selected for further analysis. These strains were grown overnight in microaerobic shake-flasks SMG, were diluted with water to an OD_660_ of 5 and eight 100 µL aliquots of each strain were distributed into a 96-well plate. From this plate, 96-well plates containing 100 µL SMG/well, containing acetic acid concentrations ranging from 8 to 15 g/L, were inoculated. Plates were sealed with a gas impermeable seal, incubated at 30 °C for 5 days without shaking, followed by measurement of OD_660_. Segregants for which the phenotype observed in the initial test was confirmed were selected for further study.

### Sequencing of selected genes in haploid segregants

Different haploid segregants derived from the cross of the evolved strains with IMK439 were grown separately in SMG and the volume of aliquot equivalent to 1 mL with an OD_660_ of 1.0 was determined for each culture. For each evolution mutant, the aliquots of all (4–9) acetic acid-tolerant segregants were pooled (with a maximum of 6 segregants per pool), centrifuged, and after removal of supernatant, resuspended in water. This procedure was also applied to the (1 or 2) sensitive segregants of each evolved strain. Genomic DNA was extracted from the resulting cell suspensions with the YeaStar™ Genomic DNA kit (Zymo Research, Irvine, CA) and used as a template for amplification of the mutated genes identified in each evolved strain (primers listed in Additional file 2). Amplified alleles were Sanger sequenced (Baseclear) and presence of the wild-type, mutated allele or both were assessed with Clone Manager (Scientific & Educational Software, Morrisville, NC).

### Reverse engineering of acetic acid tolerance

**Fig. 1:**
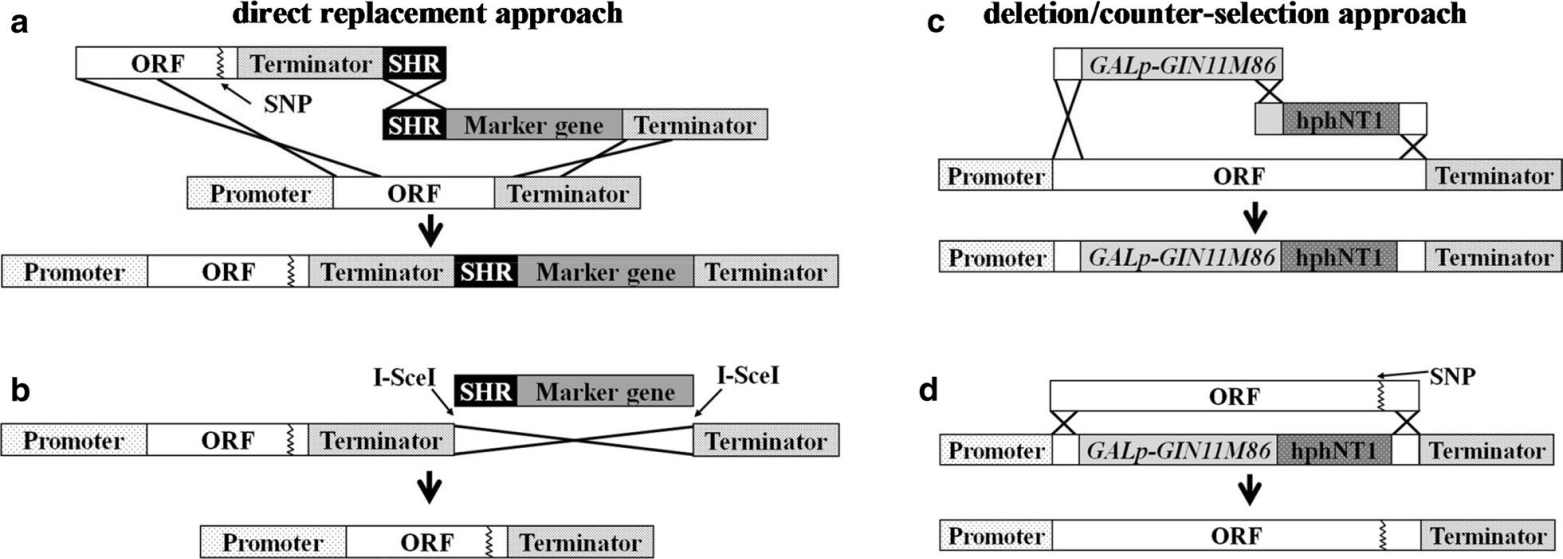
Schematic representation of the reverse engineering approach for the introduction of the mutated alleles.** a**, ** b** show the direct replacement approach:** a** two cassettes were amplified, one containing the mutated allele and its terminator region amplified from genomic DNA of the corresponding mutant, flanked downstream by the restriction site for the I-SceI endonuclease and a synthetic homologous recombination sequence (SHR) [79]. A second cassette was amplified from plasmid pUG6 [80] for kanMX and from plasmid pUG-natNT2 for natNT2 containing the marker gene flanked upstream by the same SHR sequence as the first cassette and downstream by the restriction site for the I-SceI endonuclease and by 50 bp homologous to the region immediately downstream of the ORF. Upon co-transforming the two cassettes into *S. cerevisiae*, recombination at the SHR sequence results in integration in the genome and replacement of the wild-type allele. Transformants were selected in YPD containing G418 and/or nourseothricin.** b** Strains containing the marker gene were transformed with plasmid pUDE206 [81] expressing the I-SceI endonuclease and selected on YPD agar plates containing hygromycin. I-SceI cuts upstream and downstream of the marker gene, resulting in homologous recombination of the repeated terminator region, and thereby removal of the marker. Removal of marker genes was confirmed by the absence of growth on YPD agar plates containing G418 and/or nourseothricin. For removal of pUDE206, strains containing pUDE206 were grown on YPD and colonies were isolated on YPD agar plates. Plasmid removal was confirmed by the absence of growth on YPD agar plates containing hygromycin.** c**,** d** show the deletion/counter-selection approach:** c** two cassettes were amplified, one containing the GALp-GIN11M86 gene amplified from pGG119 [49], flanked upstream by 50 bp homologous to the ORF of the gene to be deleted. A second cassette was amplified from plasmid pUG-hphNT1 [82] containing the hphNT1 marker, flanked upstream by 50 bp homologous to the GALp-GIN11M86 gene and downstream by 50 bp homologous to the ORF to be deleted. Upon co-transforming the two cassettes into *S. cerevisiae*, they recombine at the SHR sequence insert into the genome, replacing the wild-type allele.** d** The mutated ORF of the gene was amplified from genomic DNA of the corresponding mutant and was used to transform the strain containing GALp-GIN11M86. Transformants were selected on YPGal agar plates. Replacement of the deleted allele was tested by the ability of transformants to grow in YPGal, inability to grow in the presence of hygromycin, as well as by PCR

Mutated alleles identified in the acetic acid-tolerant mutants were introduced into CEN.PK113-7D via either of two approaches: (i) direct replacement of the wild-type allele with the mutated one [38] and subsequent marker removal (Fig. 1a, b), or (ii) deletion of the wild-type allele using the counter-selectable gene *GIN11M86* [49] and replacement of the deleted gene with the mutated allele (Fig. 1c, d). Mutated alleles of *ASG1* and *ADH3* were introduced via the direct replacement approach, whereas the deletion/counter-selection approach was used for those of *GIS4* and *SKS1*. Both fragment insertion and marker loss were confirmed by PCR amplification followed by fragment size analysis and the presence of the evolved allele bearing the single-nucleotide substitution was confirmed by Sanger sequencing. Cassettes and primers used for all allele-swapping experiments are provided in Additional files 3, 4 and 5.

## Results

### Inducibility of acetic acid tolerance in *S. cerevisiae*

To investigate induction of acetic acid tolerance by permissive concentrations of acetic acid, shake-flask cultures of *S. cerevisiae* CEN.PK113-7D cells were pre-grown on SMG medium pH 4.5 with or without 9 g/L acetic acid. These non-adapted and adapted cultures were used to inoculate microtiter plate cultures on SMG, supplemented with acetic acid at concentrations ranging from 0 to 15 g/L. When growth occurred, glucose was always fully consumed within 5 days. Final OD_660_, measured after 5 days, was therefore used as an indicator for biomass yield on glucose.

**Fig. 2:**
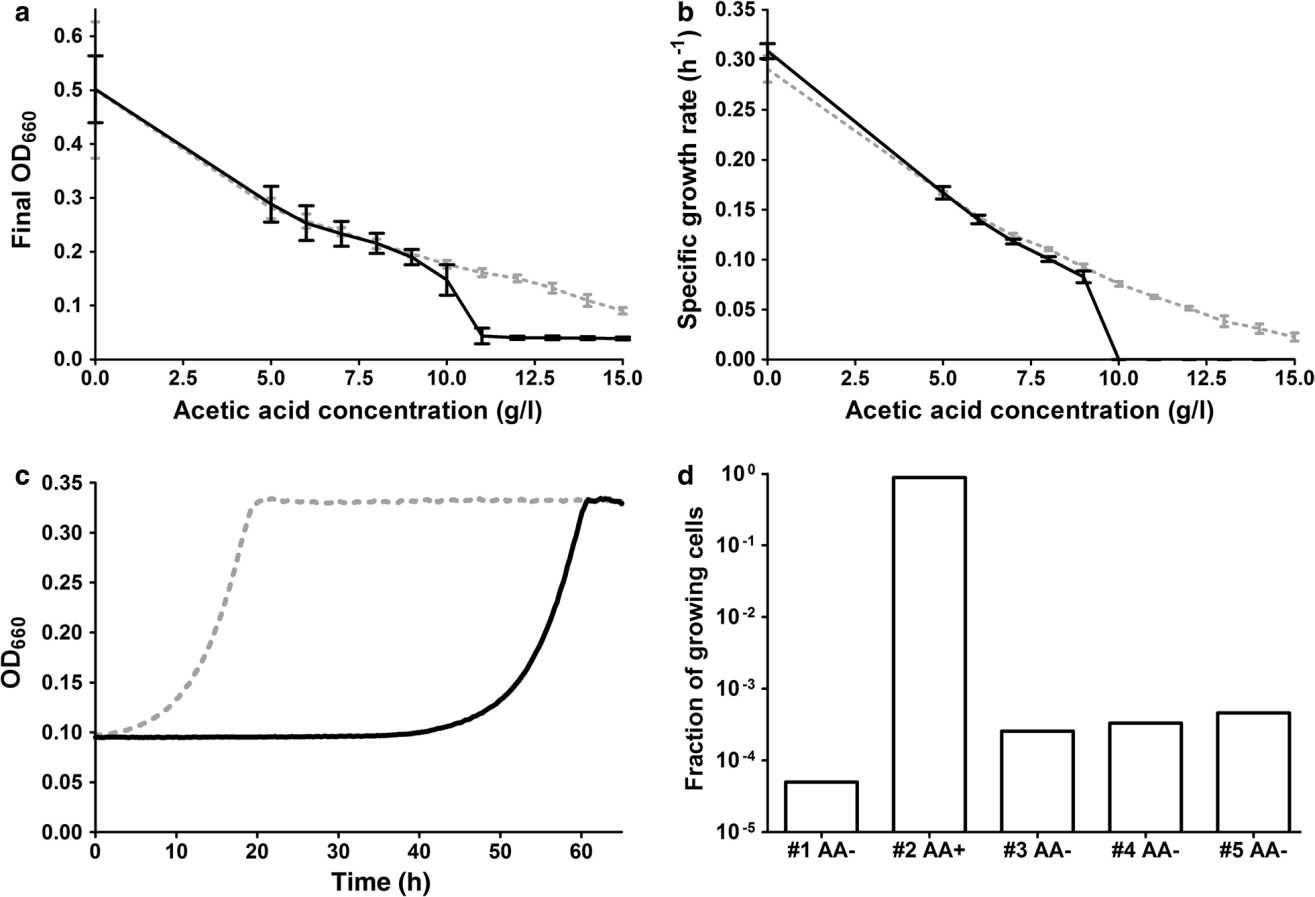
Impact of prior adaptation to acetic acid on growth in acetic acid-containing media. In all experiments, adapted precultures of *S. cerevisiae* CEN.PK113-7D were grown on SMG (pH 4.5) with 9 g/L acetic acid while non-adapted precultures were grown on SMG set at pH 6.0.** a** OD_660_ after 5 days of incubation in 96-well plates containing SMG supplemented with 0–15 g/L acetic acid and inoculated with adapted or non-adapted precultures. Data represent average and standard deviation of 32 replicate wells distributed over two independent experiments for adapted CEN.PK113-7D cells (*shown in grey*) and from 144 replicates from six independent experiments for non-adapted CEN.PK113-7D cells (*shown in black*) per acetic acid concentration. The average final OD_660_ was based on only wells in which growth occurred (final OD_660_ >0.075), except for non-adapted cells incubated with 11–15 g/L, for which the average OD_660_ of all replicates is shown (fewer than 5.7 % of wells were rejected based on this criterion).** b** Specific growth rate of CEN.PK113-7D in the same experiment. Data represent the average values and standard deviations calculated from three to six replicates distributed over four independent experiments for adapted CEN.PK113-7D cells (*shown in grey*) and five to ten replicates distributed over five independent experiments for non-adapted CEN.PK113-7D cells (*shown in black*).** c** OD_660_-based growth profiles in SMG with 9 g/L acetic acid, inoculated with acetic-adapted (*grey*) and non-adapted (*black*) precultures.** d** Fraction of stationary phase population capable of colony formation on SMG agar (pH 4.5) containing 9 g/L acetic acid during a serial transfer experiment in shake-flasks. The first flask was grown on SMG (pH 6.0) without acetic acid (referred to as #1 AA−), followed by a culture grown on SMG (pH 4.5) with 9 g/L acetic acid (referred to as #2 AA+). The three subsequent, consecutive cultures were grown on SMG (pH 6.0) without acetic acid (referred to as #3 AA−, #4 AA− and #5 AA−)

Cultures inoculated with non-adapted and adapted cells both showed a negative correlation of biomass yield and specific growth rate with acetic acid concentration (Fig. 2a, b). However, at concentrations above 10 g/L, growth was only observed in cultures inoculated with adapted cells. At acetic acid concentrations that were permissive for adapted as well as non-adapted inocula, biomass yields and specific growth rates were similar (Fig. 2a, b). Despite these similar biomass yields and specific growth rates, the adapted inocula yielded much shorter latency phases than non-adapted inocula at permissive concentrations. For example, adapted cells immediately started to grow upon transfer to fresh SMG containing 9 g/L acetic acid, and stationary phase was reached within 20 h (Fig. 2c). Non-adapted inocula only showed detectable growth after a 40-h latency phase and stationary phase was reached 20 h later. These observations are consistent with a previously described long latency phase of non-adapted cells upon transfer to acetic acid containing media, which was attributed to the observation that only a small fraction of non-adapted populations of *S. cerevisiae* cells initiated growth upon transfer to acetic acid containing media [25].

To investigate how prior adaptation to acetic acid affected the fraction of the population capable of initiating growth in acetic acid containing media, CEN.PK113-7D was grown until stationary phase in five sequential microaerobic shake-flask cultures: first in SMG medium without acetic acid, then in SMG medium with acetic acid at pH 4.5 to induce acetic acid tolerance, and then in three sequential cultures in SMG medium without acetic acid. At the end of each culture, tenfold dilutions of the cell broth were plated on agar with and without acetic acid and colony-forming units (CFU) were counted after 4–6 days of incubation to determine the fraction of growing cells. In the first culture, grown on SMG without acetic acid (pH 6.0), only 50 out of a million cells (a fraction of 0.00005) grew upon transfer to SMG agar with 9 g/L acetic acid (referred to as #1 AA−, Fig. 2d). In the stationary phase of the subsequent culture, grown on SMG and supplemented with 9 g/L acetic acid (pH 4.5), the fraction of growing cells had increased by more than 10,000-fold to 0.88 (referred to as #2 AA+ , Fig. 2d). Already in the stationary phase of the first of three subsequent shake-flasks on SMG without acetic acid (pH 6.0), this fraction had decreased to 0.00025 and did not change significantly during the two subsequent cultures in the same medium (#3 AA−, #4 AA−, #5 AA−, Fig. 2d). These results show that adaptation of *S. cerevisiae* to acetic acid induces an apparent tolerance of the overall culture, primarily by increasing the fraction of the population that is able to grow in acetic acid containing medium. However, after a single culture under non-stressed conditions, this fraction rapidly returns to within one order of magnitude of its original, non-adapted level.

### Laboratory evolution for constitutive acetic acid tolerance

**Fig. 3:**
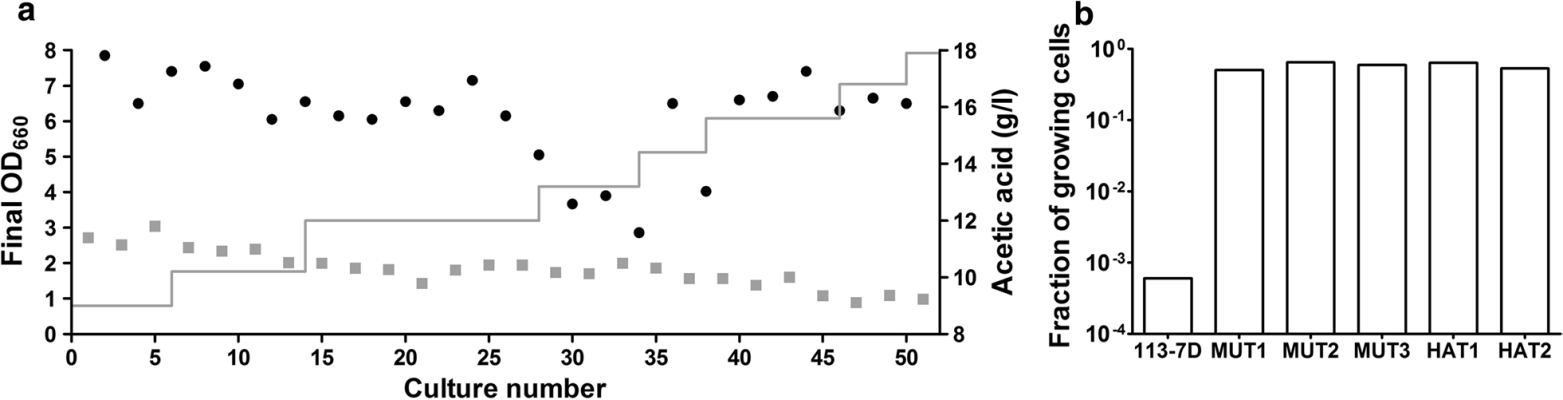
Description of laboratory evolution for constitutive acetic acid tolerance in microaerobic serum flasks and characterization of resulting mutants.** a** Laboratory evolution for constitutive acetic acid tolerance (evolution experiment MUT1). After UV mutagenesis *S. cerevisiae* CEN.PK113-7D cells were grown in a series of sequential microaerobic batch cultures, alternatingly in the presence (*grey squares*) and absence (*black circles*) of acetic acid. Over a series of 50 transfers, the acetic acid concentrations in the stressed culture cycles were progressively increased from 9 to 18 g/L (*grey line*).** b** Fraction of cells able to grow on SMG agar plates with 9 g/L acetic acid (pH 4.5) after three consecutive microaerobic batch cultures on SMG (pH 6.0) without acetic acid. The first culture was inoculated from the final serum bottle in the presence of 18 g/L acetic acid from the evolution lines of MUT1, MUT2, MUT3, HAT1 and HAT2. To mimic the growth conditions of the mutants, prior to the three cultures without acetic acid also CEN.PK113-7D was induced in SMG with 9 g/L acetic acid and subsequently grown in SMG with 18 g/L acetic acid, corresponding to the final concentration of acetic acid during the mutants evolution

The results presented above showed that induced tolerance due to preadaptation by growth in the presence of acetic acid was already lost after one shake-flask culture in the absence of added acetic acid (Fig. 2d). This observation inspired a laboratory evolution strategy based on serial transfer in microaerobic batch cultures, alternatingly grown in the presence and absence of acetic acid (Fig. 3a). The rationale of this experimental design was to confer a selective advantage to constitutively acetic acid tolerant cells that immediately and efficiently initiate growth upon transfer to acetic acid containing medium and to select against cells that cannot initiate growth in such media. Before each transfer to medium without acetic acid, cells were washed to remove acetic acid. Moreover, during the evolution cycles, cultures in non-stressed conditions were performed at pH 6.0 to reduce the impact of any acetic acid left in or produced by the cultures. Five independent parallel evolution experiments were performed with this strategy. In three of these experiments (referred to as MUT1, MUT2 and MUT3), *S. cerevisiae* CEN.PK113-7D cells were UV mutagenized prior to the first batch culture, while the other two experiments (referred to as HAT1 and HAT2) were started with non-mutagenized cells. Throughout the evolution experiments, the stationary phase OD_660_ of cultures in the presence of acetic acid was consistently lower than in the non-stressed cultures of the evolution cycles (Fig. 3a). This negative impact on biomass yield increased as the acetic acid concentration in the stressed culture cycles was progressively increased from 9 to 18 g/L during the evolution experiments (Fig. 3a). After 50 transfers, all evolved cultures exhibited microaerobic growth on glucose at pH 4.5 in the presence of 18 g/L acetic acid, indicating a markedly increased tolerance relative to non-adapted CEN.PK113-7D cells (Fig. 2a).

To investigate whether the tolerance observed in the laboratory evolution experiments was indeed constitutive, three additional transfers of all five evolved cultures were performed in SMG without acetic acid (pH 6.0). After the last culture, the fraction of cells able to grow on SMG agar plates with 9 g/L acetic acid was determined (Fig. 3b). As a reference, a culture of CEN.PK113-7D was pre-grown with 9 g/L acetic acid to induce acetic acid tolerance and subsequently transferred to new medium containing 18 g/L acetic acid prior to the determination of the fraction of growing cells after three additional cultures without acetic acid (Fig. 3b). After these three transfers, all five independently evolved cultures showed at least a 1000-fold higher fraction of the population that was able to grown on SMG agar with 9 g/L acetic acid (pH 4.5) compared to the unevolved reference strain CEN.PK113-7D (Fig. 3b).

**Fig. 4:**
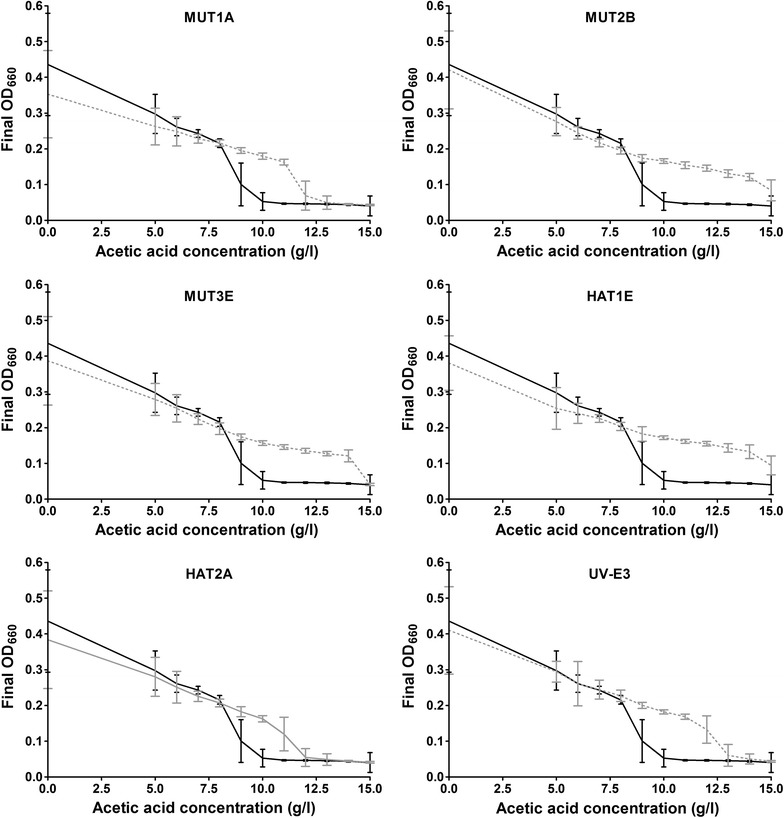
Growth of single cell isolates from five independent serial transfer experiments with* S. cerevisiae*, evolved for increased acetic acid tolerance (MUT1A, MUT2B, MUT3E, HAT1E and HAT2A) and one acetic acid tolerant strain obtained by UV mutagenesis and screening (UV-E3). Strains were grown in 96-well plates on SMG (pH 4.5) with 0–15 g/L acetic acid without prior adaptation to acetic acid. Final OD_660_ of the six mutants (*grey lines*) and of reference strain CEN.PK113-7D (*black lines*) were measured after 5 days of incubation. Data points represent average and standard deviation from 32 replicates distributed over four independent experiments for every mutant and from 48 replicates for CEN.PK113-7D distributed over six independent experiments. At high acetic acid concentrations the relatively low fraction of growing cells coupled to stochasticity of the ability to grow, results in an increased number of wells without growth, and thereby in relatively high standard deviations of the average OD_660_

From all five evolution cultures several single-cell colonies were isolated and the acetic acid tolerance of these isolates was compared with that of the unevolved reference strain CEN.PK113-7D in 96-well plates with SMG containing 0–15 g/L acetic acid. All isolates were able to grow at higher acetic acid concentrations than the reference strain (data not shown), and from each evolution culture the isolate that showed growth at the highest acetic acid concentration (MUT1A, MUT2B, MUT3E, HAT1E and HAT2A) was selected for further study (Fig. 4). The different degree of tolerance of the five single-cell isolates suggested that the genetic basis for tolerance in these strains might be different (Fig. 4). Two out of five cell lines isolated after laboratory evolution (MUT2B and HAT1E) were even able to grow at an acetic acid concentration of 15 g/L.

### Selection of a constitutively acetic acid tolerant mutant by mutagenesis and screening

In parallel to the laboratory evolution approach, a constitutively acetic acid tolerant mutant was isolated by a modified procedure for random mutagenesis and screening. Preliminary experiments based on UV mutagenesis followed by testing of random colonies for acetic acid tolerance failed to yield constitutively tolerant mutants. Indeed, we were unable to isolate mutants (based on single colonies on plates) with increased acetic acid tolerance directly after UV mutagenesis (data not shown). We assume this is due to the extremely low probability that a single cell has to resume proliferation in the presence of acetic acid, even if it contains a mutation that is beneficial for acetic acid tolerance. Therefore, after UV-irradiation, cells were first grown in SMG (pH 6.0) for 2 days without acetic acid before plating on SMG with 9 g/L acetic acid (pH 4.5). In total 40 randomly selected single-cell-colony isolates from this experiment were then pre-grown in SMG without acetic acid (pH 6.0), followed by screening of their tolerance in 96-well plates containing SMG with 9 and 10 g/L acetic acid (pH 4.5). The five UV-mutants that reached the highest OD_660_ in this screening were further characterized in 96-well plates (8 replicates per strain) containing 7, 8, 9 or 10 g/L acetic acid. In these experiments, strain UV-E3 consistently showed the highest final OD_660_. Its increased constitutive acetic acid tolerance relative to CEN.PK113-7D was confirmed by growing it in 96-well plates on SMG with acetic acid concentrations ranging between 0 and 15 g/L (Fig. 4). By allowing mutants to proliferate by prior cultivation in medium without acetic acid, the absolute number of cells harbouring specific mutations was increased. As even for beneficial mutants, only a fraction of cells is able to grow upon transfer to medium with acetic acid, the generally increased number of cells decreased the chance to lose a beneficial genotype due to single-cell stochasticity of growth. This modified procedure resulted in the selection of strain UV-E3 with improved constitutive acetic acid tolerance.

### Genomic mutations in constitutively acetic acid tolerant mutants

To identify the mutations underlying the increased constitutive acetic acid tolerance of strains MUT1A, MUT2B, MUT3E, HAT1E, HAT2A and UV-E3, their genomes were sequenced and compared to that of the reference strain CEN.PK113-7D. This comparison yielded several single-nucleotide polymorphisms (see Additional file 6), with numbers varying from 5 for UV-E3 to 21 SNPs for HAT1E. In addition, strains MUT3E and HAT1E showed copy number changes of chromosomal regions which did, however, not harbour genes with single-nucleotide changes relative to the reference strain (see Additional file 7, 8). Six genes showed single-nucleotide changes in multiple strains: *SAC6*, *EUG1*, *ASG1*, *ADH3*, *SKS1* and *GIS4* (Table 3). *SAC6* and *EUG1* carried the same mutation in all five evolved strains, but not in UV-E3, which may indicate that these two mutations arose prior to the start of the laboratory evolution experiments. In contrast, mutations in *ASG1*, *ADH3*, *SKS1* and *GIS4* differed among acetic acid tolerant strains (Table 3). *ASG1*, which encodes a transcriptional regulator involved in stress response [59, 60], showed different mutations in four strains. In all three strains that contained (different) mutations in *ADH3*, which encodes a mitochondrial alcohol dehydrogenase involved in NADH shuttling to the cytosol under anaerobic conditions [61, 62], mutations in *ASG1* were also found. Similarly, mutations in *SKS1*, which encodes a protein kinase involved in adaptation to low glucose concentrations [63], were only found in combination with mutated alleles of both *ASG1* and *ADH3*. *GIS4*, which encodes a protein linked to ion homeostasis and to glucose derepression of *SUC2* [64, 65], was mutated in strains MUT3E and UV-E3.

**Table 3:** Mutations present in multiple evolved* S. cerevisiae* strains and genetic analysis of their relevance for acetic acid tolerance

Gene	Strain	SNP	Nucleotide in THS	Nucleotide in SHS
ASG1	MUT1A	G1248**A**	**A**	G
	MUT2B	G1248**T**	**T**	G
	HAT1E	A1979**G**	**G**	A
	HAT2A	G2881**C**	**C**	G
ADH3	MUT1A	G416**T**	**T**	G
	MUT2B	T966**G**	T-**G**	T
	HAT2A	T201**A**	**A**	T
SKS1	MUT2B	G821**T**	**T**	G
	HAT2A	C617**A**	C-**A**	nd
GIS4	MUT3E	G1322**C**	**C**	G
	UV-E3	G295**A**	nd	nd
SAC6	MUT1A	G491**A**	**A**	G
	MUT2B	G491**A**	nd	**A**
	MUT3E	G491**A**	G-**A**	G
	HAT1E	G491**A**	G	**A**
	HAT2A	G491**A**	G-**A**	**A**
EUG1	MUT1A	G-113**T**	G	G-**T**
	MUT2B	G-113**T**	G-**T**	**T**
	MUT3E	G-113**T**	G	**T**
	HAT1E	G-113**T**	G-**T**	G
	HAT2A	G-113**T**	**T**	**T**

Mutations were identified by comparing whole-genome sequencing data of single-cell isolates MUT1A, MUT2B, MUT3E, HAT1E, HAT2A and UV-E3 to the genome sequence of* S. cerevisiae* CEN.PK113-7D [44]. Mutations shown occur in at least two strains. In addition to the nucleotide changes, the distribution of the SNP among tolerant haploid segregants (THS) and the sensitive haploid segregants (SHS) of a diploid, generated by mating the tolerant strains with* S. cerevisiae* IMK439, are shown. Nucleotides present in the evolved strains are bold and underlined. Two nucleotides separated by a dash indicate presence of both mutated and reference-strain alleles in pooled THS or SHS.* nd* no sequence data obtained

### Genetic analysis for identification of causal mutations

The observation that *ASG1*, *ADH3*, *SKS1*, *GIS4*, *SAC6* and *EUG1* were mutated in multiple acetic acid tolerant strains suggested that they might contribute to this acquired phenotype. To further investigate a possible role of these genes in tolerance, a genetic analysis was performed. The strains obtained from the laboratory evolution experiments (but not the UV-mutagenized strain UV-E3), were mated with IMK439, a *MATαura3Δ::*KanMX strain congenic with CEN.PK113-7D. The resulting diploids were sporulated after which haploid segregants were tested for acetic acid tolerance. Subsequently, the presence or absence of mutated alleles was tested in haploid segregants with the same tolerance level as the mutants (tolerant segregants) and as CEN.PK113-7D (sensitive segregants). To this end, genomic DNA of all tolerant segregants derived from the same diploid was pooled (Additional file 9) and fragments containing the relevant SNPs in *ASG1*, *ADH3*, *SKS1*, *GIS4*, *SAC6* and *EUG1* were PCR amplified. The same was done for a pool of sensitive haploid segregants derived from each diploid. Sanger sequencing revealed that mutated and wild-type alleles of *SAC6* and *EUG1* were randomly distributed in the population of tolerant and sensitive haploid segregants from all five evolved strains (Table 3). Conversely, mutated alleles of *ASG1* and *GIS4* were exclusively found in tolerant segregants, while all sensitive segregants tested contained the wild-type allele (Table 3). It was therefore concluded that mutations in *ASG1* and in *GIS4*, but not those in *SAC6* and *EUG1*, contributed to the acquired acetic acid tolerance. For both *ADH3* and *SKS1*, all the sensitive haploid segregants contained the wild-type alleles, but the tolerant haploid segregants did not exclusively contain mutated alleles. The pooled tolerant segregants from strain MUT2B contained both wild-type and mutated alleles of *ADH3* and only the mutated allele of *SKS1*. Oppositely, for HAT2A both wild-type and mutated alleles of *SKS1* and only the mutated allele of *ADH3* were identified in the pooled tolerant segregants. This suggests that mutations in *ADH3* and *SKS1* contributed to acetic acid tolerance, but their impact may not be additive.

### Reverse engineering of constitutive acetic acid tolerance

**Fig. 5:**
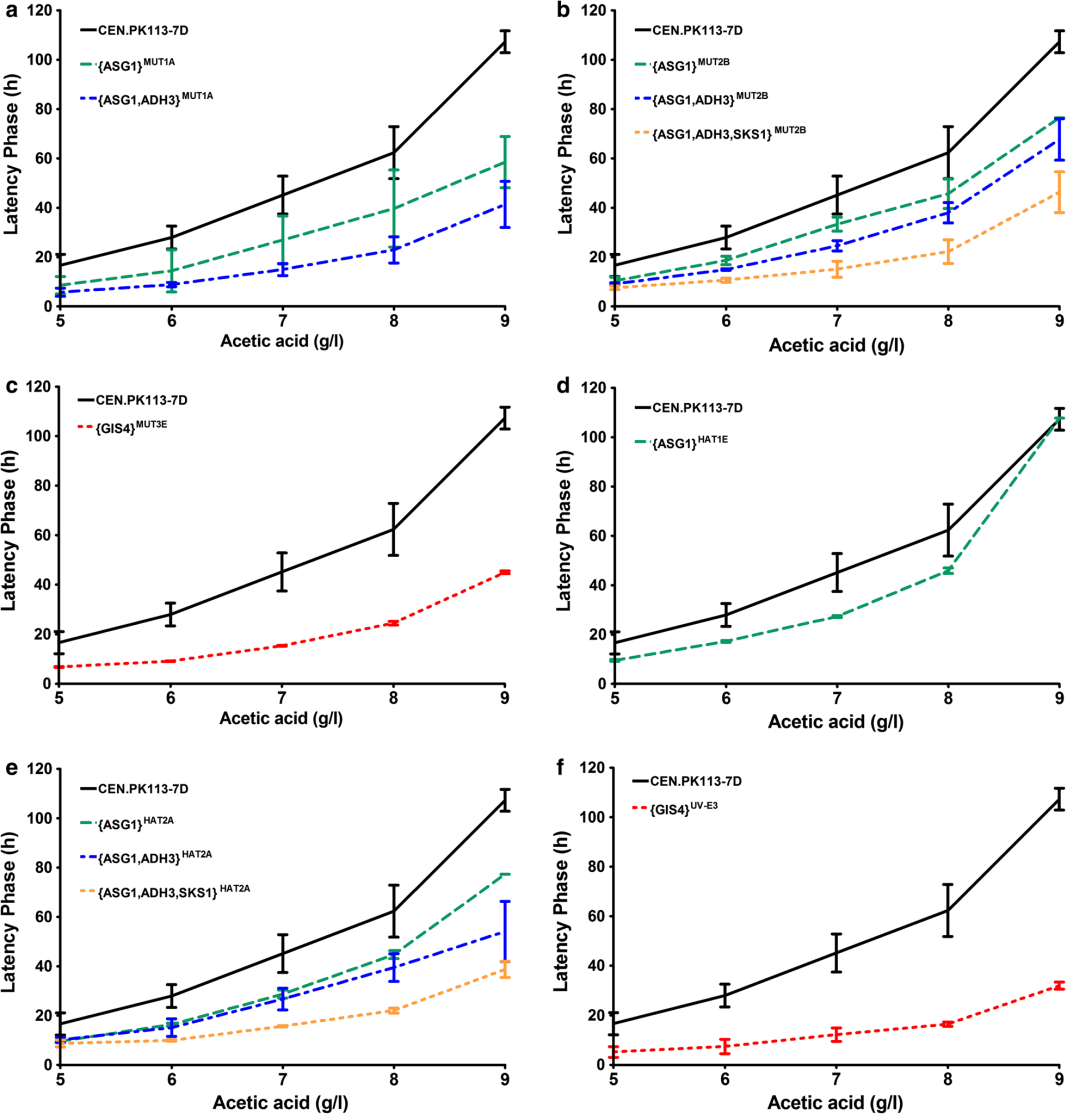
Latency phase of engineered strains and CEN.PK113-7D upon inoculation of non-adapted cells in media containing acetic acid. After pre-cultivation in SMG without acetic acid (pH 6.0), cells were inoculated in 96-well plates containing SMG with acetic acid (pH 4.5, 5–9 g/L acetic acid) at an initial OD_660_ of 0.1. Sealed plates were incubated at 30 °C and at 400 rpm, and the OD_660_ was recorded every 15 min. For each acetic acid concentration, length of the latency phase was defined as the time required to reach an OD_660_ of 0.12. Data points represent average and standard deviation of two to eight replicates. Wells that did not show growth after 5 days (OD_660_ <0.12) were eliminated from the analysis (15 out of 312 for the reverse engineered strains and 44 out of 156 for CEN.PK113-7D).** a** Engineered strains expressing ASG1 and ADH3 alleles from MUT1A.** b** Engineered strains expressing ASG1, ADH3 and SKS1 alleles from MUT2B.** c** Engineered strain expressing the GIS4 allele from MUT3E.** d** Engineered strain expressing the ASG1 allele from HAT1E.** e** Engineered strains expressing ASG1, ADH3 and SKS1 alleles from HAT2A.** f** Engineered strains expressing the GIS4 allele from UV-E3

The causal mutations identified in the genetic analysis were introduced in *S. cerevisiae* strain CEN.PK113-7D to further investigate their contribution to constitutive acetic acid tolerance. In the process, no alterations beyond the introduction of the corresponding single-nucleotide were introduced. The sequential introduction of mutations was prioritised based on the number of strains in which the specific gene was mutated and the causality for acetic acid tolerance in the haploid segregation studies. Acetic acid tolerance of the resulting reverse engineered strains was first analysed by comparing the latency phase upon inoculation of non-adapted cells in SMG containing 5–9 g/L acetic acid. Separate introduction of the four mutated *ASG1* alleles in CEN.PK113-7D resulted in a significantly shorter latency phase at high concentrations of acetic acid (Fig. 5a, b, d and e). This latency phase was further reduced when additionally a mutated *ADH3* allele from the same evolved strain was introduced (Fig. 5a, b, e). In strains already carrying mutated *ASG1* and *ADH3* alleles from MUT2B and HAT2A, the additional introduction of mutated alleles of *SKS1* led to an even further shortening of the latency phase (Fig. 5b, e). Introduction of only the mutated *GIS4* alleles from MUT3E and UV-E3 in CEN.PK113-7D resulted a substantially reduced latency phase, comparable to that observed upon combined introduction of mutated *ASG1*, *ADH3* and *SKS1* alleles (Fig. 5c, f).

**Fig. 6:**
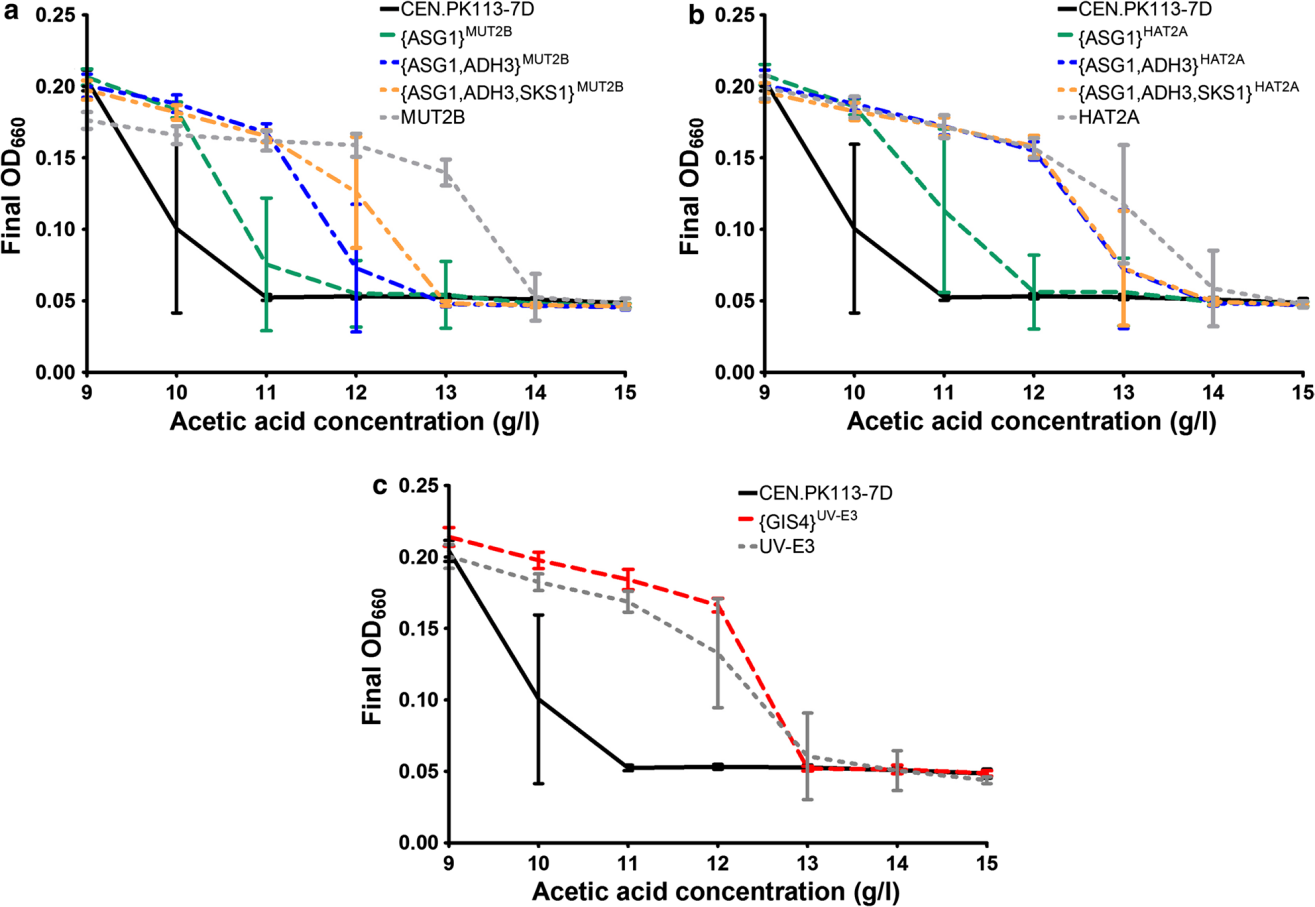
Acetic acid tolerance of engineered* S. cerevisiae* strains and CEN.PK113-7D upon transfer of non-adapted cells to acetic acid-containing media. Reverse engineered strains containing mutated alleles from evolved and/or mutagenized strains MUT2B, HAT2A and UV-E3 were pre-grown in SMG without acetic acid (pH 6.0), and inoculated at an initial OD_660_ of 0.1 in 96-well plates containing SMG with 9–15 g/L acetic acid (pH 4.5). Final OD_660_ values were recorded after 5 days of incubation. Data points represent average and standard deviation of 16 replicate wells for engineered strains and of 32 replicate wells for the reference strain* S. cerevisiae* CEN.PK113-7D.** a** Engineered strains expressing ASG1, ADH3 and SKS1 alleles from MUT2B.** b** Engineered strains expressing ASG1, ADH3 and SKS1 alleles from HAT2A.** c** Engineered strain expressing the GIS4 allele from UV-E3

Acetic acid tolerance of the reverse engineered strains was further analysed by comparing the OD_660_ reached after 5 days of cultivation on SMG at acetic acid concentrations ranging from 9 to 15 g/L. As in the latency phase assays, an additive beneficial effect of the mutated alleles of *ASG1*, *ADH3* and *SKS1* was observed (Fig. 6a, b). While only some of the replicate cultures of CEN.PK113-7D grew at 10 g/L acetic acid, all replicates of the strains with only a mutated *ASG1* allele grew at this concentration, and some also grew at 11 g/L. Strains that additionally carried a mutated *ADH3* allele from MUT2B or HAT2A, grew up to acetic acid concentrations of 12 and 13 g/L, respectively. Additional introduction of the mutated *SKS1* allele from MUT2B resulted in an increased number of replicates in which growth occurred at these high acetic acid concentrations. A strain expressing only the mutated *GIS4* allele from UV-E3 also grew at higher acetic acid concentrations than CEN.PK113-7D, up to a concentration of 12 g/L (Fig. 6c). Some replicate cultures of the original evolved strains MUT2B and HAT2A showed growth up to an acetic acid concentration of 14 g/L, a concentration at which none of the reverse engineered strains grew. This observation indicates that the mutated alleles of *ASG1*, *ADH3* and *SKS1* explain most, but not all of the acquired acetic acid tolerance of these evolved strains. The remaining difference between the tolerance of the reconstructed and the evolved strains may be due to additional mutations in the evolved strains, including *SAC6* and *EUG1* that were not investigated for causality in this study.

## Discussion

Acetic acid is a key inhibitor of yeast performance in lignocellulosic hydrolysates, but can also be produced by *S. cerevisiae* during cultivation under anaerobic conditions [46]. Wild-type strains of *S. cerevisiae* have evolved an inducible tolerance against this organic acid, in which the *HAA1* regulon, which includes the *TPO2*- and *TPO3*-encoded membrane transporters, plays an important role [66]. In a previous study, in which *S. cerevisiae* was evolved in the laboratory through prolonged growth in the presence of progressively increasing acetic acid concentrations, the evolved cultures acquired an increased tolerance which, however, required prior induction by acetic acid [41]. At first glance, such a ‘hyper-inducible’ acetic acid tolerance phenotype can be interpreted as reflecting an increased maximum expression level of the natural, inducible acetic acid tolerance mechanisms by all cells in the culture [67]. Recent observations, however, suggest that the situation may be more complicated. Pronounced heterogeneity in the adaptation of genetically homogeneous microbial populations to changing environmental conditions has been described for many micro-organisms and culture parameters [68–70]. Recently, such a heterogeneous response was also observed upon the exposure of non-stressed *S. cerevisiae* cultures to acetic acid stress, which showed that only a small fraction of the population resumed growth [20, 25, 71]. For a given *S. cerevisiae* strain, the fraction of a non-stressed and non-induced population able to grow at a specific acetic acid concentration and pH was shown to be constant and an important determinant of acetic acid tolerance [25].

In this study, we show that preadaptation in the presence of acetic acid strongly increases the fraction of the population that is able to grow in acetic acid containing medium. In particular, preadaptation strongly reduced the duration of the latency phase upon transfer to acetic acid containing media, while growth rate and biomass yield remain unaltered. Preadaptation and culture heterogeneity are not explicitly included in other recent studies on selection and/or construction of acetic acid tolerant *S. cerevisiae* strains [30–35]. However, in those studies, pre-cultivation typically involved batch cultivation at low pH in glucose-containing media. It is conceivable that in these cultures, acetic acid produced by the yeast cultures was sufficient to induce acetic acid tolerance, an effect known as hormesis [72]. In contrast, in the current industrial practice of second-generation bioethanol production, yeast biomass is typically pre-grown in a separate aerobic, sugar-limited fed-batch process. Conditions in this biomass propagation phase are especially chosen to optimize biomass yield, and therefore, to minimize formation of by-products such as ethanol and acetic acid. To ensure an efficient start of anaerobic fermentation of the subsequent acetic acid-containing lignocellulosic hydrolysates, inhibitor tolerance should already be fully expressed during the preceding ‘non-stressed’ biomass propagation phase.

The observation that acetic acid adaptation of *S. cerevisiae* cultures was rapidly lost upon growth in the absence of acetic acid at pH 6.0, provided the basis for a simple evolutionary engineering strategy. Prolonged cultivation in five independent evolution experiments with alternating stressed and non-stressed culture cycles reproducibly yielded evolved *S. cerevisiae* cultures that, after pre-cultivation in the absence of acetic acid, could immediately initiate growth at high acetic acid concentrations. As a side effect, this strategy adds a selective pressure against mutations that in addition to benefits for acetic acid tolerance results in decreased fitness during the off-phase, which would potentially decrease the industrial applicability. This simple ‘on–off’ strategy is likely to be applicable for the selection of constitutive tolerance to a variety of chemical or physical stresses, especially in situations where micro-organisms already harbour native, inducible tolerance mechanisms.

After initial identification of mutations by whole-genome resequencing of independent evolution lines and classical genetic analysis, causality of SNPs in *ASG1*, *ADH3, SKS1* and *GIS4* for acetic acid tolerance was confirmed by reverse engineering of an unevolved strain. Interestingly, three of these genes (*ADH3*, *SKS1* and *GIS4*) were not previously associated with acetic acid tolerance in genome-wide expression studies [27, 28], screening of deletion libraries [73] or in a recent quantitative-trait-loci analysis [30]. This difference can probably be explained from the focus, in this study, on constitutive tolerance rather than on the maximum permissive acetic acid concentration. Indeed, the acquired acetic acid tolerance of the evolved cultures was predominantly due to a strong increase of the fraction of the population that was able to initiate growth upon a transfer to acetic acid containing conditions.

Based on sequence homology and limited functional analysis, Asg1, Sks1 and Gis4 have been implicated in regulation, but their precise functions in acetic acid tolerance remain to be elucidated. Protein sequence information available from the *Saccharomyces* genome database [74] indicates that the mutations identified in this study did not affect amino-acid residues that are known or predicted to be post-translationally modified or to be in narrowly defined functional domains. However, deletion of *ASG1* in *S. cerevisiae* was previously shown to result in increased susceptibility to acetic acid. Indeed, in *Candida glabrata* deletion of *ASG1* resulted in reduced plasma membrane H^+^-ATPase activity, an inability to regulate intracellular pH and leading to growth inhibition in acidic environments. Identification of causal mutations in *ADH3*, which encodes the main mitochondrial isoenzyme of NAD^+^-dependent alcohol dehydrogenase, is puzzling, since it is difficult to imagine how its catalytic role could influence acetic acid tolerance [62]. A recently reported role of Adh3 in cold tolerance of *Saccharomyces* yeasts [75] was tentatively attributed to its role in cellular redox balancing. Clearly, further molecular studies are required to elucidate how the four genes identified in this study affect the distribution of acetic acid-tolerant and -sensitive cells in cultures that have not previously been exposed to acetic acid. Recently, a link between the cytosolic pH and stochasticity of growth in the presence of acetic acid was identified [26]. The fact that stochasticity of growth is determined at the single cell level precludes elucidation of the underlying mechanisms at a population level. In this light, recent developments on genomics of single cells in combination with microfluidic devices might prove to be valuable to investigate this cell-to-cell heterogeneity as well as the mechanisms of identified beneficial mutations [76–78]. Even without full characterization of their mechanistic roles in acetic acid tolerance, the genes and alleles identified in this study provide leads for the improvement of industrial strains of *S. cerevisiae*.

## Conclusions

Alternating growth in the presence and absence of acetic acid provided a selective pressure for mutants with constitutive acetic acid tolerance, as reflected by an increased fraction of the population that was able to initiate growth in the presence of high concentrations of acetic acid without prior adaptation to this stressor. Awareness of the importance of culture heterogeneity and preadaptation proved valuable in the design of optimized laboratory evolution as well as mutagenesis strategies. Similar approaches are likely to be applicable to improve other characteristics of industrial micro-organisms that are inducible and/or involve culture heterogeneity. Mutations in three genes (*ASG1*, *ADH3* and *SKS1*) were identified that individually, as well as synergistically, contributed to acetic acid tolerance. Additionally, alleles of *GIS4* were identified that caused a similar level of constitutive acetic acid tolerance. Both strain improvement approaches, as well as the underlying mutations, can be used for industrial strain engineering and thereby stimulate further development of second generation processes for the production of fuels and chemicals.

## Additional files

10.1186/s13068-016-0583-1 Sequences bioproject accession numbers.
10.1186/s13068-016-0583-1 Primers used to sequence the SNPs.
10.1186/s13068-016-0583-1 Primers used for the reverse engineering of mutated alleles.
10.1186/s13068-016-0583-1 Primers, cassettes and SHR used for direct replacement approach.
10.1186/s13068-016-0583-1 Primers, cassettes and SHR used for deletion/counter-selection approach.
10.1186/s13068-016-0583-1 List of SNPs in all strains.
10.1186/s13068-016-0583-1 Copy number analysis genomes MUT3E.
10.1186/s13068-016-0583-1 Copy number analysis genomes HAT1E.
10.1186/s13068-016-0583-1 Haploid segregant strains.
