# Supplementary material for: A new laboratory evolution approach to select for constitutive acetic acid tolerance in Saccharomyces cerevisiae and identification of causal mutations
Source: Biotechnol Biofuels. 2016 Aug 12;9:173. doi: 10.1186/s13068-016-0583-1 (PMC4983051; doi:10.1186/s13068-016-0583-1)
Supplement: Supplementary file 1 — 10.1186/s13068-016-0583-1 Sequences bioproject accession numbers. [file 13068_2016_583_MOESM1_ESM.docx]

Additional file 2: Accession numbers of the short reads obtained by whole genome sequencing of the mutants. Note that the sequence reads of UV-E3 have been deposited as mutant E3 due to a nomenclature change.

| Bioproject | PRJNA313456/SRP070976 |  |  |
| --- | --- | --- | --- |
| Strain | Biosample | experiment | run |
| UV-E3 (aka mutant E3) | SAMN04523136/SRS1315526 | SRX1605922 | SRR3195521 |
| MUT3E | SAMN04523135/SRS1315525 | SRX1605921 | SRR3195516 |
| MUT2B | SAMN04523134/SRS1315524 | SRX1605920 | SRR3195513 |
| MUT1A | SAMN04523133/SRS1315523 | SRX1605918 | SRR3195511 |
| HAT2A | SAMN04523116/SRS1315521 | SRX1605916 | SRR3195492 |
|  |  |  | SRR3195496 |
| HAT1E | SAMN04523115/SRS1315520 | SRX1605914 | SRR3195490 |
