# Supplementary material for: A new laboratory evolution approach to select for constitutive acetic acid tolerance in Saccharomyces cerevisiae and identification of causal mutations
Source: Biotechnol Biofuels. 2016 Aug 12;9:173. doi: 10.1186/s13068-016-0583-1 (PMC4983051; doi:10.1186/s13068-016-0583-1)
Supplement: Supplementary file 2 — 10.1186/s13068-016-0583-1 Primers used to sequence the SNPs. [file 13068_2016_583_MOESM2_ESM.docx]

Additional file 3: Sequence of the primers used for the amplification and sequencing of the regions around the SNPs obtained in the evolution and UV-mutants.

| Target gene | Mutated allele | Primer name | Sequence 5' - 3' |
| --- | --- | --- | --- |
| *ASG1* | MUT1A, MUT2B | ASG1CON-MUT2B1A-F | ATCGCTGTGGGAGCATTATTC |
|  |  | ASG1CON-MUT2B1A-R | CTTTGGGCAACGAATCCAAC |
|  | HAT1E | ASG1CON-HAT1E-F | GTGGCTCGAACGGTTATTAAG |
|  |  | ASG1CON-HAT1E-R | TGATCTTGCTGTTGTGCTTC |
|  | HAT2A | ASG1CON-HAT2B-2-F | GGCGACATCCCTACTAACAG |
|  |  | ASG1CON-HAT2B-2-R | AGCAGGCCTTGGTTTATG |
| *ADH3* | MUT1A, HAT2A | ADH3CON-HAT2BMUT1A-F | AGGAACACTCGCTTTATCTC |
|  |  | ADH3CON-HAT2BMUT1A-R | CCCATCGCAGTTGCATATTG |
|  | MUT2B | ADH3CON-MUT2B-F | TGCGATGGGTTACAGAGTTC |
|  |  | ADH3CON-MUT2B-R | CGTTCTATGTGTCGACGGAAG |
| *SKS1* | MUT2B, HAT2A | SKS1CON-HAT2BMUT2B-F | ACCACACTACAGGGAGATTG |
|  |  | SKS1CON-HAT2BMUT2B-R | GCTGCTGTTGTGCTTGTTCC |
| *GIS4* | MUT3E | GIS4CON-MUT3E-F | GGTGCAGGCAGAAACATTAC |
|  |  | GIS4CON-MUT3E-R | TGTTTGCCGGAGTTTGTCAC |
|  | UV-E3 | GIS4CON-KO-A-F | GGTGCAGGCAGAAACATTAC |
|  |  | GIS4CON-Mutant_e3-R | AGTTCGGGTAGCTGTTGAAG |
| *EUG1* | MUT1A, MUT2B, MUT3E,  HAT1E, HAT2A | EUG1CON-F | AGCCATTGGCAATGGATGAC |
|  |  | EUG1CON-R | GACGAGGACTAACGGATGAG |
| *SAC6* |  | SAC6CON-F | AAGCTCTAGAAGCGGTGTCC |
|  |  | SAC6CON-R | ACCCAGGATCAAATGCTCTC |
