# Supplementary material for: A new laboratory evolution approach to select for constitutive acetic acid tolerance in Saccharomyces cerevisiae and identification of causal mutations
Source: Biotechnol Biofuels. 2016 Aug 12;9:173. doi: 10.1186/s13068-016-0583-1 (PMC4983051; doi:10.1186/s13068-016-0583-1)
Supplement: Supplementary file 3 — 10.1186/s13068-016-0583-1 Primers used for the reverse engineering of mutated alleles. [file 13068_2016_583_MOESM3_ESM.docx]

Additional file 4: Sequence of the primers used for the reverse engineering of the mutated genes. The function of these primers and the templates they were used on can be found in additional files 5 and 6. SHR sequences are shown in bold and the I-SceI endonuclease sites are highlighted in yellow.

| Primer nameP Primer name | Sequence 5' - 3' |
| --- | --- |
| B-Amds-F | **CACCTTTCGAGAGGACGATGCCCGTGTCTAAATGATTCGACCAGCCTAAGAATGTTCAAC**CAGCTGAAGCTTCGTACGC |
| Amds-ASG1-R | AAATACACACATAAACGTTTTAAGCTTATGTTCAGCAAGTGCTGTAGCCAAGTTACGCTAGGGATAACAGGGTAATATAGCACTATAGGGAGACCGGCAG |
| ASG1up-F | TTGGCCAGTGAGTTGAGTTC |
| ASG1-B-R | **GTTGAACATTCTTAGGCTGGTCGAATCATTTAGACACGGGCATCGTCCTCTCGAAAGGTG**AGTTACGCTAGGGATAACAGGGTAATATAGGGACGGTGATTATGACCTTG |
| ASG1check-F | AGCTATTTGGGAAATACCTACC |
| ASG1check-R | TACGACAAGGCACAACGTTC |
| ADH3-Amds-F | TCTGTTCACAGTTAAAACTAGGAATAGTATAGTCATAAGTTAACACCATCAGTTACGCTAGGGATAACAGGGTAATATAGCAGCTGAAGCTTCGTACGC |
| A-Amds-R | **GTGCCTATTGATGATCTGGCGGAATGTCTGCCGTGCCATAGCCATGCCTTCACATATAGT**CACTATAGGGAGACCGGCAG |
| A-ADH3-F | **ACTATATGTGAAGGCATGGCTATGGCACGGCAGACATTCCGCCAGATCATCAATAGGCAC**AGTTACGCTAGGGATAACAGGGTAATATAGAGCCCATAGGGTCCTTTAAC |
| ADH3Down2-R | ATGGCTACGTGTTGCTGTTG |
| ADH3check-F | CGGTCCCGCAGAATTAATAG |
| ADH3check-R | GACACGCCTGGTGAACAATG |
| GIN11-R | CGTTAAAGCTGGTCGACGGTATC |
| KanMX-GIN11-F | CTGCAGGAATTCGATATCAAGCTTATCGATACCGTCGACCAGCTTTAACGCAGCTGAAGCTTCGTACGC |
| SKS1-GIN11-F | GCTCCGCATTAGATCACTGTCATCGTCTCGGTATATATCATTGTGATATCGGTGCCGTAAAGCACTAAATCG |
| KanMX-SKS1-R | TATCGTTAGCAAAATGTTGAAATGTGTTATCCTCCTTCTGGTGAGCTTTTCACTATAGGGAGACCGGCAG |
| SKS1CON-KO-A-F | GGACGAAGACGAAGAAAGAC |
| SKS1CON-KO-D-R | CATTCAAATGCGCCGTCG |
| SKS1CON-HAT2BMUT2B-F | ACCACACTACAGGGAGATTG |
| SKS1CON-HAT2BMUT2B-R | GCTGCTGTTGTGCTTGTTCC |
| GIS4-GIN11-F | CAGGCCAAACAAGAAGATTTTGTTAGTTTCCATCCCAGAGAATGTTCATGGGTGCCGTAAAGCACTAAATCG |
| KanMX-GIS4-R | TCCTGAATTAATATAGATTGCAAAACTAGCCTAAAATACCCTAGAGAATCCACTATAGGGAGACCGGCAG |
| GIS4CON-KO-A-F | GGTGCAGGCAGAAACATTAC |
| GIS4CON-KO-D-R | GGGTTGTCATCAATGCAGAAG |
| GIS4CON-MUT3E-R | TGTTTGCCGGAGTTTGTCAC |
