# Supplementary material for: A new laboratory evolution approach to select for constitutive acetic acid tolerance in Saccharomyces cerevisiae and identification of causal mutations
Source: Biotechnol Biofuels. 2016 Aug 12;9:173. doi: 10.1186/s13068-016-0583-1 (PMC4983051; doi:10.1186/s13068-016-0583-1)
Supplement: Supplementary file 4 — 10.1186/s13068-016-0583-1 Primers, cassettes and SHR used for direct replacement approach. [file 13068_2016_583_MOESM4_ESM.docx]

Additional file 5: Primers, cassettes and SHR sequences used to reverse engineer *ASG1* and *ADH3* via the direct replacement approach. From left to right: target gene for reverse engineering, cassettes used for the reverse engineering, primers used to amplify the cassettes, Synthetic Homologous Recombination (SHR) sequences used to promote recombination between the two cassettes (see reference 79), and primers for the confirmation of the correct marker integration.

| Target gene | Amplification of the cassettes | | | | Primers confirmation marker insertion/removal |
| --- | --- | --- | --- | --- | --- |
|  | Cassette | Template | Primers | SHR |  |
| *ASG1* | natNT2 marker | pUG-natNT2 | B-Amds-F/  Amds-ASG1-R | B | ASG1check-F/ ASG1check-R |
|  | Mutated *ASG1* allele | gDNA of the respective mutant | ASG1up-F/  ASG1-B-R |  |  |
| *ADH3* | kanMX marker | pUG6 | ADH3-Amds-F/  A-Amds-R | A | ADH3check-F/ ADH3check-R |
|  | Mutated *ADH3* allele | gDNA of the respective mutant | A-ADH3-F/  ADH3Down2-R |  |  |
