# Supplementary material for: A new laboratory evolution approach to select for constitutive acetic acid tolerance in Saccharomyces cerevisiae and identification of causal mutations
Source: Biotechnol Biofuels. 2016 Aug 12;9:173. doi: 10.1186/s13068-016-0583-1 (PMC4983051; doi:10.1186/s13068-016-0583-1)
Supplement: Supplementary file 5 — 10.1186/s13068-016-0583-1 Primers, cassettes and SHR used for deletion/counter-selection approach. [file 13068_2016_583_MOESM5_ESM.docx]

Additional file 6: Primers, cassettes and SHR sequences used to reverse engineer *SKS1* and *GIS4* via the deletion/counter-selection approach. From left to right: target gene for reverse engineering, cassettes used for the reverse engineering, primers used to amplify the cassettes, and primers for the confirmation of the deletion/counter-selection.

| Target gene | Cassette amplification | | | Primers confirmation deletion/counter-selection |
| --- | --- | --- | --- | --- |
|  | Cassettes | Template | Primers |  |
| *SKS1* | *GALp-GIN11M86* | pGG119 | SKS1-GIN11-F/  GIN11-R | SKS1CON-HAT2BMUT2B-F/  SKS1CON-HAT2BMUT2B-R |
|  | hphNT1 marker | pUG-hphNT1 | KanMX-GIN11-F/  KanMX-SKS1-R |  |
|  | Mutated *SKS1* allele | gDNA of the respective mutant | SKS1CON-KO-A-F/  SKS1CON-KO-D-R |  |
| *GIS4* | *GALp-GIN11M86* | pGG119 | GIS4-GIN11-F/  GIN11-R | GIS4CON-KO-A-F/  GIS4CON-MUT3E-R |
|  | hphNT1 marker | pUG-hphNT1 | KanMX-GIN11-F/  KanMX-GIS4-R |  |
|  | Mutated GIS4 allele | gDNA of the respective mutant | GIS4CON-KO-A-F/  GIS4CON-KO-D-R |  |
