# Supplementary material for: A new laboratory evolution approach to select for constitutive acetic acid tolerance in Saccharomyces cerevisiae and identification of causal mutations
Source: Biotechnol Biofuels. 2016 Aug 12;9:173. doi: 10.1186/s13068-016-0583-1 (PMC4983051; doi:10.1186/s13068-016-0583-1)
Supplement: Supplementary file 6 — 10.1186/s13068-016-0583-1 List of SNPs in all strains. [file 13068_2016_583_MOESM6_ESM.docx]

**Additional file 7:** Single nucleotide variation identified in the evolved strains MUT1A, MUT2B, MUT3E, HAT1E, HAT2A and in the mutagenized strain UV-E3. Mutations upstream of the genes are indicated by a negative base pair number. Mutations downstream of the genes are indicated as such. * Indicates the introduction of an early stop codon.

| **Strain** | **Gene** | **Nucleotide change** | **Aminoacid change** |
| --- | --- | --- | --- |
|  |  |  |  |
| MUT1A |  |  |  |
|  | *SIT4* | G473T | G158V |
|  | *SAC6* | G491A | A164V |
|  | *POP7* | C301T | D101N |
|  | *ASG1* | G1248A | M416I |
|  | *ADH3* | G416T | C139F |
|  | *COS9* | A494C | K165T |
|  | *PTR2* | G1191T | L397F |
|  | *HCR1* | G739A | Q247* |
|  | *SCD5* | G2150T | S717* |
|  | *YDL063C* | T1272C | L424L |
|  | *YDR169C-A* | G159A | G53G |
|  | *TRZ1* | G93A | F31F |
|  | *ARG3* | C408T | I136I |
|  | *EUG1* | G-113T | None |
|  | *YGR127W* | C (42 bp downstream) T | None |
|  |  |  |  |
| MUT2B |  |  |  |
|  | *ARO4* | C43T | V15I |
|  | *ASG1* | G1248T | M416I |
|  | *GIP2* | T53A | K18I |
|  | *RAS1* | C461A | A154E |
|  | *SAC6* | G491A | A164V |
|  | *SKS1* | G821T | P274Q |
|  | *VPS16* | C1037T | A346V |
|  | *YLR278C* | G1819T | Q607K |
|  | *ADH3* | T966G | Y322* |
|  | *EMP70* | G639A | Y213Y |
|  | *YLR445W* | T315C | P105P |
|  | *ADH4* | C-401T | None |
|  | *DSE3* | G-114C | None |
|  | *EUG1* | G-113T | None |
|  | *ADY2* | C (190 bp downstream) G | None |
|  | *GCD1* | A (77 bp downstream) G | None |
|  |  |  |  |
| MUT3E |  |  |  |
|  | *ADE12* | G709A | D237N |
|  | *GIS4* | G1322C | S441* |
|  | *YLR072W* | A1882T | R628* |
|  | *IXR1* | G320T | S107* |
|  | *PCL10* | A765C | V255V |
|  | *EUG1* | G-113T | None |

| **Strain** | **Allele** | **Nucleotide change** | **Aminoacid change** |
| --- | --- | --- | --- |
|  |  |  |  |
| HAT1E |  |  |  |
|  | *NUP145* | C432A | N144K |
|  | *MSH5* | T2048C | L683S |
|  | *SAC6* | G491A | A164V |
|  | *CCR4* | G323T | S108Y |
|  | *ASG1* | A1979G | E660G |
|  | *OCA5* | G218T | P73Q |
|  | *RIC1* | T645A | L215F |
|  | *YKR051W* | C449A | P150Q |
|  | *POL1* | G3224T | R1075L |
|  | *AVT4* | C1695A | F565L |
|  | *YDR333C* | C2149A | E717* |
|  | *CDC1* | T1452C | F484F |
|  | *LCB4* | T1173C | R391R |
|  | *FZF1* | A-152T | None |
|  | *EUG1* | G-113T | None |
|  | *IFA38* | T-234G | None |
|  | *TMA108* | C-245T | None |
|  | *OM45* | A-376T | None |
|  | *COX19* | A-146T | None |
|  | *COX19* | C-147T | None |
|  | *ACO2* | A-394C | None |
|  |  |  |  |
| HAT2A |  |  |  |
|  | *SAC6* | G491A | A164V |
|  | *ASG1* | G2881C | G961R |
|  | *LEU3* | C839A | P280H |
|  | *SKS1* | C617A | G206V |
|  | *ADH3* | T201A | Y67* |
|  | *ISD11* | T-465G | None |
|  | *EUG1* | G-113T | None |
|  | *PDI1* | G-584C | None |
|  | *STE6* | T-521C | None |
|  | *STE12* | G (85 bp downstream) A | None |
|  |  |  |  |
| UV-E3 |  |  |  |
|  | *CLD1* | G664A | E222K |
|  | *YFR045W* | G265A | A89T |
|  | *CHR3_JIGSAW_84352988_GENE* | G148A | E50K |
|  | *GIS4* | G295A | Q99* |
|  | *PXL1* | C528T | F176F |
