# Supplementary material for: A new laboratory evolution approach to select for constitutive acetic acid tolerance in Saccharomyces cerevisiae and identification of causal mutations
Source: Biotechnol Biofuels. 2016 Aug 12;9:173. doi: 10.1186/s13068-016-0583-1 (PMC4983051; doi:10.1186/s13068-016-0583-1)
Supplement: Supplementary file 7 — 10.1186/s13068-016-0583-1 Copy number analysis genomes MUT3E. [file 13068_2016_583_MOESM7_ESM.docx]

**Additional file 8:** Regions in the genome of MUT3E showing Copy Number Variations respectively to the genome of CENPK113-7D.

| Chromosome number | Start | Stop | Contig length | Copy Number MUT3E | Copy Number  CEN.PK113-7D |
| --- | --- | --- | --- | --- | --- |
| 01 | 46254 | 66159 | 19906 | 2 | 1 |
|  | 66060 | 67466 | 1407 | 2 | 1 |
|  | 67453 | 70901 | 3449 | 1 | 1 |
|  | 127697 | 128715 | 1019 | 2 | 1 |
|  | 128746 | 131048 | 2303 | 2 | 1 |
|  | 131051 | 139156 | 8106 | 2 | 1 |
|  | 139257 | 139765 | 509 | 1 | 1 |
|  | 139766 | 140563 | 798 | 4 | 2 |
| 03 | 1198 | 4628 | 3431 | 2 | 1 |
|  | 4629 | 5411 | 783 | 3 | 2 |
|  | 5679 | 6222 | 544 | 3 | 2 |
|  | 6290 | 9223 | 2934 | 3 | 2 |
|  | 9269 | 11225 | 1957 | 3 | 2 |
|  | 11226 | 11508 | 283 | 1 | 1 |
|  | 11509 | 12238 | 730 | 2 | 2 |
|  | 14019 | 52900 | 38882 | 2 | 1 |
|  | 52972 | 54889 | 1918 | 2 | 1 |
|  | 54950 | 57046 | 2097 | 2 | 1 |
|  | 57047 | 82463 | 25417 | 2 | 1 |
|  | 83039 | 84072 | 1034 | 2 | 1 |
| 04 | 1101676 | 1105906 | 4231 | 2 | 1 |
|  | 1105906 | 1106401 | 496 | 2 | 1 |
|  | 1106432 | 1135674 | 29243 | 2 | 1 |
|  | 1135676 | 1150834 | 15159 | 2 | 1 |
|  | 1151318 | 1154292 | 2975 | 2 | 1 |
|  | 1156007 | 1159684 | 3678 | 2 | 1 |
|  | 1161399 | 1163160 | 1762 | 2 | 1 |
|  | 1163310 | 1163915 | 606 | 3 | 1 |
|  | 1164022 | 1175378 | 11357 | 2 | 1 |
|  | 1175538 | 1193143 | 17606 | 2 | 1 |
|  | 1193207 | 1201738 | 8532 | 2 | 1 |
|  | 1201738 | 1206696 | 4959 | 2 | 1 |
| 08 | 12561 | 35753 | 23193 | 2 | 1 |
|  | 35851 | 52590 | 16740 | 2 | 1 |
|  | 52591 | 63906 | 11316 | 2 | 1 |
|  | 63939 | 85280 | 21342 | 2 | 1 |
|  | 85906 | 91398 | 5493 | 1 | 1 |
|  | 104831 | 105088 | 258 | 2 | 2 |
|  | 105089 | 115805 | 10717 | 2 | 1 |
|  | 115817 | 116098 | 282 | 1 | 1 |
|  | 116435 | 119463 | 3029 | 2 | 1 |
|  | 119464 | 133007 | 13544 | 2 | 1 |
