# Supplementary material for: A new laboratory evolution approach to select for constitutive acetic acid tolerance in Saccharomyces cerevisiae and identification of causal mutations
Source: Biotechnol Biofuels. 2016 Aug 12;9:173. doi: 10.1186/s13068-016-0583-1 (PMC4983051; doi:10.1186/s13068-016-0583-1)
Supplement: Supplementary file 8 — 10.1186/s13068-016-0583-1 Copy number analysis genomes HAT1E. [file 13068_2016_583_MOESM8_ESM.docx]

**Additional file 9:** Regions in the genome of HAT1E showing Copy Number Variations respectively to the genome of CENPK113-7D.

| Chromosome number  Chr01 | Start | Stop | Contig length | Copy Number HAT1E | Copy Number CEN.PK113-7D |
| --- | --- | --- | --- | --- | --- |
| 02 | 8328 | 9106 | 779 | 2 | 1 |
|  | 9676 | 19064 | 9389 | 2 | 1 |
|  | 19065 | 29673 | 10609 | 2 | 1 |
| 04 | 1212613 | 1243221 | 30609 | 2 | 1 |
|  | 1243222 | 1245753 | 2532 | 4 | 2 |
|  | 1245754 | 1301920 | 56167 | 2 | 1 |
|  | 1302003 | 1305621 | 3619 | 2 | 1 |
|  | 1305705 | 1307687 | 1983 | 2 | 1 |
|  | 1308586 | 1352458 | 43873 | 2 | 1 |
| 07 | 736419 | 739126 | 2708 | 2 | 1 |
|  | 739217 | 763901 | 24685 | 2 | 1 |
|  | 763905 | 764974 | 1070 | 3 | 2 |
|  | 764975 | 778925 | 13951 | 2 | 1 |
| 09 | 325102 | 325745 | 644 | 2 | 1 |
|  | 325826 | 336275 | 10450 | 2 | 1 |
|  | 336421 | 339225 | 2805 | 2 | 1 |
|  | 339227 | 370377 | 31151 | 2 | 1 |
|  | 370425 | 389254 | 18830 | 2 | 1 |
|  | 389255 | 390713 | 1459 | 2 | 1 |
|  | 390846 | 391110 | 265 | 4 | 2 |
|  | 392665 | 406765 | 14101 | 2 | 1 |
|  | 406773 | 431759 | 24987 | 2 | 1 |
|  | 434278 | 437540 | 3263 | 2 | 1 |
