# Supplementary material for: A new laboratory evolution approach to select for constitutive acetic acid tolerance in Saccharomyces cerevisiae and identification of causal mutations
Source: Biotechnol Biofuels. 2016 Aug 12;9:173. doi: 10.1186/s13068-016-0583-1 (PMC4983051; doi:10.1186/s13068-016-0583-1)
Supplement: Supplementary file 9 — 10.1186/s13068-016-0583-1 Haploid segregant strains. [file 13068_2016_583_MOESM9_ESM.docx]

Additional file 1: Acetic-acid tolerant and sensitive haploid segregants derived from the evolution mutants and IMK439 by crossing and sporulation. These strains were used for the haploid allele segregation studies.

| Strain | Relevant description | Source |
| --- | --- | --- |
| M1A-P1E9. | Tolerant haploid segregant of MUT1A-D | This study |
| M1A-P1G4. | Tolerant haploid segregant of MUT1A-D | This study |
| M1A-P1G7. | Tolerant haploid segregant of MUT1A-D | This study |
| M1A-P2C10. | Tolerant haploid segregant of MUT1A-D | This study |
| M1A-P1B11. | Sensitive haploid segregant of MUT1A-D | This study |
| M1A-P1H8. | Sensitive haploid segregant of MUT1A-D | This study |
| M2B-P1A6 | Tolerant haploid segregant of MUT2B-D | This study |
| M2B-P1A7 | Tolerant haploid segregant of MUT2B-D | This study |
| M2B-P1A12 | Tolerant haploid segregant of MUT2B-D | This study |
| M2B-P1F10 | Tolerant haploid segregant of MUT2B-D | This study |
| M2B-P2C4 | Tolerant haploid segregant of MUT2B-D | This study |
| M2B-P2H8 | Tolerant haploid segregant of MUT2B-D | This study |
| M2B-P2D6 | Sensitive haploid segregant of MUT2B-D | This study |
| M3E-P1C7 | Tolerant haploid segregant of MUT3E-D | This study |
| M3E-P1F6 | Tolerant haploid segregant of MUT3E-D | This study |
| M3E-P1F8 | Tolerant haploid segregant of MUT3E-D | This study |
| M3E-P1G10 | Tolerant haploid segregant of MUT3E-D | This study |
| M3E-P2C8 | Sensitive haploid segregant of MUT3E-D | This study |
| H1E-P1C6 | Tolerant haploid segregant of HAT1E-D | This study |
| H1E-P1C8 | Tolerant haploid segregant of HAT1E-D | This study |
| H1E-P1G5 | Tolerant haploid segregant of HAT1E-D | This study |
| H1E-P1G8 | Tolerant haploid segregant of HAT1E-D | This study |
| H1E-P1H5 | Tolerant haploid segregant of HAT1E-D | This study |
| H1E-P1H6 | Tolerant haploid segregant of HAT1E-D | This study |
| H1E-P2C7 | Tolerant haploid segregant of HAT1E-D | This study |
| H1E-P2D5 | Tolerant haploid segregant of HAT1E-D | This study |
| H1E-P2E9 | Tolerant haploid segregant of HAT1E-D | This study |
| H1E-P1D11 | Sensitive haploid segregant of HAT1E-D | This study |
| H2A-P2D12 | Tolerant haploid segregant of HAT2A-D | This study |
| H2A-P1A10 | Tolerant haploid segregant of HAT2A-D | This study |
| H2A-PC24 | Tolerant haploid segregant of HAT2A-D | This study |
| H2A-P2C8 | Tolerant haploid segregant of HAT2A-D | This study |
| H2A-P1A12 | Sensitive haploid segregant of HAT2A-D | This study |
